# Catalyst for Industrial‐Scale Seawater Electrolysis: Inhibit Active Metal Dissolution and Chlorine Corrosion

**DOI:** 10.1002/advs.202514301

**Published:** 2025-09-16

**Authors:** Peng Wang, Jie Zheng, Yuyang Li, Qiaofu Shi, Jun Zhang, Yong Wan, Mang Niu, Yusuke Yamauchi, Yun‐Ze Long

**Affiliations:** ^1^ Shandong Key Laboratory of Medical and Health Textile Materials College of Physics Qingdao University Qingdao 266071 P. R. China; ^2^ Industrial Research Institute of Nonwovens & Technical Textiles Shandong Center for Engineered Nonwovens (SCEN) College of Textiles Clothing Qingdao University Qingdao 266071 P. R. China; ^3^ State Key Laboratory of Bio‐fibers and Eco‐textiles Institute of Biochemical Engineering College of Materials Science and Engineering Qingdao University Qingdao 266071 P. R. China; ^4^ School of Chemical Engineering The University of Queensland Brisbane QLD 4072 Australia; ^5^ Department of Materials Process Engineering Graduate School of Engineering Nagoya University Nagoya 464‐8603 Japan

**Keywords:** alkaline seawater electrolysis, anti‐chlorine corrosion, hydrogen production, industrial‐scale

## Abstract

Alkaline seawater electrolysis is a promising technology for sustainable green hydrogen production. However, active metal dissolution and chlorine‐induced corrosion during long‐term, industrial‐scale operation pose critical challenges to catalyst stability. Here, a surface engineering strategy is reported using phytic acid (PA) as a molecular “armor” to construct highly durable oxygen evolution reaction (OER) catalysts. Comprehensive characterization and density functional theory (DFT) calculations reveal that surface modification with PO_4_^3−^ groups not only facilitates surface reconstruction to form NiOOH active sites, but also optimizes the adsorption–desorption dynamics of key reaction intermediates, thereby enhancing catalytic performance. Importantly, the PO_4_^3−^ layer suppresses the adsorption of chloride ions at active sites, significantly improving corrosion resistance under harsh seawater conditions. As a result, the catalyst delivers a current density of 100 mA cm^−2^ at a low overpotential of 208 mV in alkaline seawater, maintaining stable performance over 1500 h. When integrated as the anode in a proton exchange membrane electrolyzer, it supports operation at 1 A cm^−2^ with a cell voltage of only 2.18 V, exhibiting no performance degradation over 500 h.

## Graphical abstract

Alkaline seawater electrolysis represents a promising approach for sustainable green hydrogen production. Nevertheless, catalyst stability remains a critical challenge due to active metal dissolution and chlorine‐induced corrosion during prolonged industrial‐scale operation. Here, a surface engineering strategy is presented that employs phytic acid (PA) as a molecular ‘armor’ to effectively prevent reactive metal dissolution and chlorine‐induced corrosion.

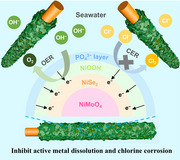

## 1 Introduction

Direct seawater electrolysis powered by renewable electricity, such as wind and solar power, offers a sustainable approach for large‐scale production of green hydrogen.^[^ ^1^, ^2^, ^3^ ^]^ However, the complex composition of seawater, especially the chloride ion (Cl^−^), poses a huge challenge to the stability of electrocatalysts for anodic oxygen evolution reaction (OER).^[^ ^4^, ^5^ ^]^ Specifically, the chlorine evolution reaction (CER), which competes with OER, produces corrosive ions and harmful gases (such as ClO^−^, HOCl, and Cl_2_), leading to catalyst corrosion and thereby reducing catalytic efficiency and long‐term stability.^[^ ^6^, ^7^ ^]^ From a reaction kinetics perspective, in the alkaline electrolyte, the reaction potential of OER is 490 mV lower than that of CER.^[^ ^8^, ^9^ ^]^ Therefore, in alkaline seawater media at industrial‐level current density, the development of OER electrocatalysts with a kinetic overpotential of less than 490 mV has attracted extensive attention. Regrettably, the activity and durability of the catalyst are seriously affected by chlorine corrosion and the poisoning of metal active sites by chloride ions during the continuous electrolysis process.^[^ ^10^, ^11^, ^12^ ^]^

Nanomaterial catalysts, such as transition metal oxides,^[^ ^13^ ^]^ nitrides,^[^ ^14^ ^]^ sulfides,^[^ ^15^ ^]^ phosphides,^[^ ^16^ ^]^ and selenides^[^ ^17^ ^]^ exhibit superior OER activity in alkaline seawater electrolysis for hydrogen production. Among these materials, transition metal selenides (TMSs) have emerged as promising candidates for alkaline OER catalysts owing to their tunable band gaps, electronic structures, and coordination environments.^[^ ^18^, ^19^, ^20^ ^]^ However, TMSs are prone to the dissolution of the Se element during the electrochemical cycle process, resulting in poor catalyst stability, which poses a great challenge to industrial‐level seawater electrolysis for hydrogen production. The OER catalytic stability of TMSs can be effectively improved to a certain extent through lattice strain engineering,^[^ ^21^, ^22^ ^]^ high entropy effect,^[^ ^23^ ^]^ and organic selenium ligands.^[^ ^24^ ^]^ However, the inevitable reconstruction of selenide and the leaching of Se at high current density remain significant challenges.^[^ ^25^ ^]^ In this context, rational utilization of the reconstructed active sites on the catalyst surface, along with surface modification of the active centers, offers a promising strategy to enhance catalytic activity and stability.^[^ ^26^, ^27^, ^28^ ^]^ Phosphate (PO_4_^3−^) group, as a kind of hard Lewis base, can act as a proton acceptor and provide electron pairs, which can accelerate the proton‐coupled electron transfer of interfacial water molecules.^[^ ^29^ ^]^ Doping catalysts with PO_4_^3−^ group has been shown to significantly enhance catalytic activity and stability in seawater electrolysis for hydrogen production.^[^ ^30^, ^31^, ^32^ ^]^ Compared with conventional phosphating agents (e.g., heavy metal‐containing phosphates), phytic acid (PA), a natural organic polyphosphate containing six PO_4_^3−^ groups, is abundant, inexpensive, and environmentally benign. In addition, PA exhibits strong electronegativity and metal‐chelating ability, allowing it to form stable coordination bonds with metal ions in the catalyst.^[^ ^33^ ^]^ These properties endow the catalyst with excellent structural stability and resistance to chlorine corrosion during seawater electrolysis.

Herein, we have dexterously designed a nickel‐based selenide (NiMoO_4_@NiSe_2_‐PA) modified with a surface amorphous PO_4_^3−^ layer to achieve industrial‐scale current density and highly stable alkaline seawater OER performance. A series of characterizations and theoretical calculations indicate that the surface PO_4_^3−^ groups coordinate the electronic states, optimizing the electronic structure of the catalyst. In addition, the surface PO_4_^3−^ groups accelerated the in situ reconstruction of Ni to generate NiOOH active sites and promote the catalytic activity. More importantly, the PO_4_^3−^ groups inhibit the adsorption of chloride ions on the metal active sites, and dynamically repel Cl^−^ ions during seawater electrolysis for hydrogen production, thus exhibiting excellent corrosion resistance. The electrochemical test results show that NiMoO_4_@NiSe_2_‐PA can achieve a current density of 100 mA cm^−2^ with an overpotential of only 208 mV in alkaline seawater and maintain long‐term durability of over 1500 h. In addition, a proton exchange membrane electrolyzer assembled with NiMoO_4_@NiSe_2_‐PA as the anode requires only a low cell voltage of 2.18 V to reach a current density of 1 A cm^−2^ and shows no degradation within over 500 h. This study not only resolves the problem of metal dissolution in selenide electrochemical processes but also provides a concise and effective strategy for the rational design of highly active and corrosion‐resistant industrial‐scale seawater electrolysis catalysts.

## 2 Results and Discussion

### 2.1 Synthesis and Characterization of Electrocatalyst

**Figure 1:**
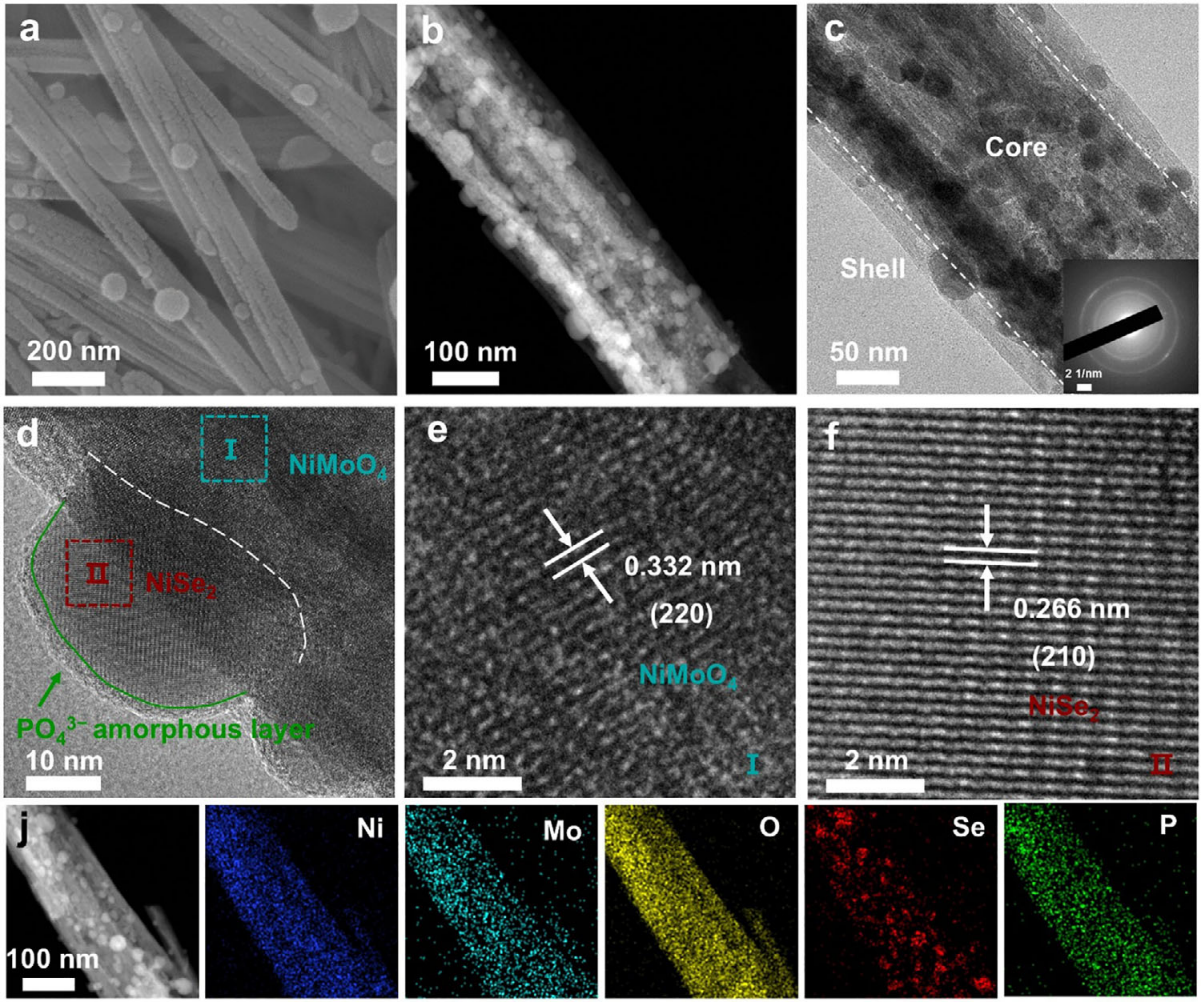
Structure characterization of the NiMoO_4_@NiSe_2_‐PA electrocatalyst. a) SEM image of the NiMoO_4_@NiSe_2_‐PA electrocatalyst. b) HAADF‐STEM image of the NiMoO_4_@NiSe_2_‐PA nanowire. c) TEM image and SAED pattern (insert) of the NiMoO_4_@NiSe_2_‐PA core‐shell nanowire. d–f) HRTEM images of the NiMoO_4_@NiSe_2_‐PA nanowire. j) HAADF‐STEM image of NiMoO_4_@NiSe_2_‐PA and corresponding EDS element mappings of Ni, Mo, O, Se, and P.

NiMoO_4_ nanowire arrays were grown in situ on 3D porous Ni foam (NF) skeleton by the hydrothermal method (Figure S1, Supporting Information). Subsequently, the NiMoO_4_@NiSe_2_ heterostructure was further grown on the surface of the nanowire using the chemical vapor deposition method (Figure S2, Supporting Information). Finally, the selenized samples were self‐assembled in PA solution to prepare NiMoO_4_@NiSe_2_‐PA electrocatalyst with surface PO_4_^3−^ modification. The scanning electron microscope (SEM) image shows that NiMoO_4_@NiSe_2_‐PA has a rough surface with numerous porous nanosheets (**Figure** **1**a). The high‐angle annular dark field scanning transmission electron microscope (HAADF‐STEM) image clearly shows the porous nanosheet structure of NiMoO_4_@NiSe_2_‐PA, which facilitates the permeation of electrolyte during seawater electrolysis and provides a larger specific surface area (Figure 1b). The transmission electron microscope (TEM) image further confirms the core‐shell structure with an ultrathin nanosheet shell (Figure 1c). The selective area electron diffraction (SAED) image of NiMoO_4_@NiSe_2_‐PA shows a series of diffraction rings (Figure 1c insert), revealing its polycrystalline structure.^[^ ^34^ ^]^ To study the fine structure of the core‐shell nanowire, NiMoO_4_@NiSe_2_‐PA was tested using high‐resolution TEM (HRTEM). As shown in Figure 1d, the core of the NiMoO_4_@NiSe_2_‐PA nanowire is identified as the NiMoO_4_ phase based on its lattice fringe spacing. The lattice spacing of 0.332 nm corresponds to the (220) plane of NiMoO_4_ (PDF#45‐0142) (Figure 1e).^[^ ^18^ ^]^ Notably, the shell of the nanowires displays a crystalline‐amorphous structure (Figure S3, Supporting Information). Numerous NiSe_2_ nanoparticles are distributed on the amorphous shell with a lattice spacing of 0.266 nm, corresponding to the (210) plane (PDF#88‐1711) (Figure 1f). In addition, the surface of NiSe_2_ was covered with an amorphous PO_4_^3−^ layer (≈3 nm thick), confirming the successful assembly of PO_4_^3−^ groups onto the surface of NiMoO_4_@NiSe_2_‐PA electrocatalyst. The energy dispersive X‐ray spectroscopy (EDS) mapping analysis revealed that Ni, Mo, O, and P elements were uniformly distributed on the NiMoO_4_@NiSe_2_‐PA nanowire, with a P content of ≈1.73 wt.% (Figure 1j; Figure S4, Supporting Information). However, the elemental mapping of Se reveals significant aggregation within the nanoparticles, corresponding to the NiSe_2_ phase in the shell, which is further confirmed by the linear elemental distribution (Figure S5, Supporting Information).

**Figure 2:**
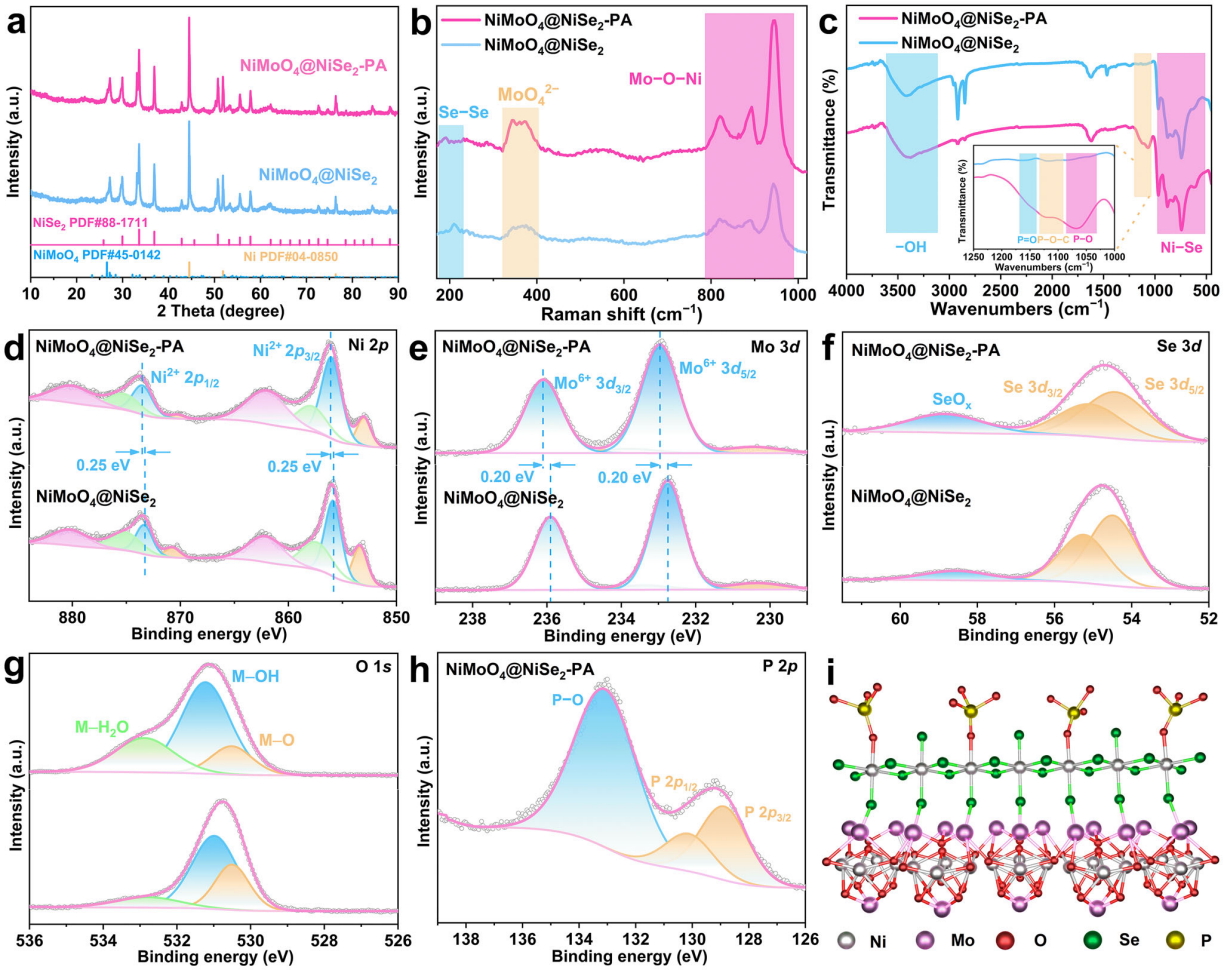
Crystal structure and surface chemical characterization of the NiMoO_4_@NiSe_2_‐PA electrocatalyst. a) XRD patterns, b) Raman spectra, and c) FTIR spectra of the NiMoO_4_@NiSe_2_ and NiMoO_4_@NiSe_2_‐PA. XPS spectra of the elements d) Ni *2p*, e) Mo *3d*, f) Se *3d*, g) O *1s*, and h) P *2p* in NiMoO_4_@NiSe_2_ and NiMoO_4_@NiSe_2_‐PA. i) Coordination structure model of the NiMoO_4_@NiSe_2_‐PA.

The crystal structure of NiMoO_4_@NiSe_2_‐PA was analyzed using X‐ray diffraction (XRD). As shown in **Figure** **2**a, the surface modification with the PO_4_^3−^ layer does not alter the crystal structure of NiMoO_4_@NiSe_2_. XRD patterns show that the NiMoO_4_@NiSe_2_‐PA is composed of standard cubic phase NiSe_2_ crystal structure (JCPDS No. 88–1711) and monoclinic phase NiMoO_4_ crystal structure (JCPDS No. 45–0142),^[^ ^35^, ^36^ ^]^ which is consistent with the TEM results. The chemical structure of NiMoO_4_@NiSe_2_‐PA was further characterized using Raman spectroscopy. As shown in Figure 2b, the characteristic peaks of 820, 890, and 942 are attributable to the Mo−O−Ni metal oxygen band of the precursor NiMoO_4_, and the characteristic peak near 350 cm^−1^ is assignable to the MoO_4_^2−^ band.^[^ ^37^, ^38^ ^]^ Furthermore, the characteristic peaks at 190 and 215.2 cm^−1^ are attributable to the vibrational mode (E_g_) and stretching mode (E_g_) of the Se−Se bond of NiSe_2_.^[^ ^39^ ^]^ The results indicate that the introduction of the surface amorphous PO_4_^3−^ layer does not disrupt the chemical coordination within the crystal structure. To further confirm the presence of the PO_4_^3−^ groups in the NiMoO_4_@NiSe_2_‐PA electrocatalyst, qualitative analysis was conducted using Fourier transform infrared (FTIR) spectroscopy. As shown in Figure 2c, the vibrational band at 500–900 cm^−1^ is attributable to the characteristic peak vibrational mode of the Ni−Se bond.^[^ ^40^ ^]^ Compared with NiMoO_4_@NiSe_2_, the bands at 1055, 1115, and 1152 cm^−1^ of NiMoO_4_@NiSe_2_‐PA are attributable to the stretching vibrations of the P−O, P−O−C, and P═O bonds, respectively, which demonstrates the successful introduction of the PO_4_^3−^ groups.^[^ ^29^, ^41^ ^]^

X‐ray photoelectron spectroscopy (XPS) was employed to investigate the effect of surface PO_4_^3−^ group modification on the surface chemical state and electronic structure of NiMoO_4_@NiSe_2_ catalyst. The XPS survey spectrum confirmed the coexistence of Ni, Mo, O, Se, and P elements in NiMoO_4_@NiSe_2_‐PA, which is consistent with the EDS results (Figure S6, Supporting Information). As shown in Figure 2d, the high‐resolution XPS spectrum of Ni *2p* for NiMoO_4_@NiSe_2_‐PA and NiMoO_4_@NiSe_2_ exhibits two distinct peaks at 855.9 and 873.5 eV, attributed to Ni^2+^ *2p_3/2_* and Ni^2+^ *2p_1/2_*, respectively.^[^ ^42^ ^]^ In addition, the peak at 852.9 eV corresponds to the characteristic peak of the NF substrate.^[^ ^43^ ^]^ Notably, the Ni *2p* characteristic peak of NiMoO_4_@NiSe_2_‐PA with surface PO_4_^3−^ groups modification showed a shift (≈0.25 eV) toward higher binding energy, indicating a decrease in the electron density near the Ni atom.^[^ ^44^ ^]^ The Mo *3d* XPS spectrum (Figure 2e) shows two typical peaks at 232.9 and 236.1 eV, corresponding to Mo^4+^ *3d_5/2_* and Mo^4+^ *3d_3/2_*, respectively.^[^ ^37^ ^]^ Compared with NiMoO_4_@NiSe_2_, the characteristic peak of Mo^4+^ *3d* also showed a positive shift (≈0.20 eV), indicating that the modification of the surface PO_4_^3−^ groups caused a change in the electronic structure of NiMoO_4_@NiSe_2_‐PA. The two characteristic peaks of 54.4 and 55.2 eV in the XPS spectrum of Se 3d are attributable to Se *3d_5/2_* and Se *3d_3/2_* (Figure 2f), indicating that Se in NiSe_2_ exists as Se^2−^. The characteristic peak at 58.9 eV is due to SeO_x_ species, resulting from the partial oxidation of Se on the surface of NiSe_2_.^[^ ^45^ ^]^ The O *1s* XPS spectrum (Figure 2g) has three distinct characteristic peaks at 530.5, 531.2, and 532.9 eV, corresponding to lattice oxygen (M−O), adsorbed hydroxyl groups (M−OH), and adsorbed water molecules (M−H_2_O), respectively.^[^ ^46^ ^]^ The high‐resolution P *2p* XPS spectrum of NiMoO_4_@NiSe_2_‐PA (Figure 2h) shows a prominent peak at 133.1 eV, attributed to the P−O bond, further confirming the presence of surface PO_4_^3−^ groups. The two weaker peaks at 128.9 and 130.2 eV are assigned to P *2p_3/2_* and P *2p_1/2_*, suggesting that the PO_4_^3−^ groups on the surface of NiMoO_4_@NiSe_2_‐PA form M−P bonds with the outer metal atoms.^[^ ^47^ ^]^

From the series of characterization results, the coordination structure model of NiMoO_4_@NiSe_2_‐PA is shown in Figure 2i. The abundant PO_4_^3−^ groups on the surface of the core‐shell nanowires firmly chelate with Ni in the NiSe_2_ shell, thereby ensuring the stability of the surface PO_4_^3−^ groups during the electrochemical reactions. Therefore, the modification of the surface PO_4_^3−^ groups not only optimizes the coordination structure but also effectively stabilizes the active site.

### 2.2 Alkaline Seawater Electrolysis Performance

**Figure 3:**
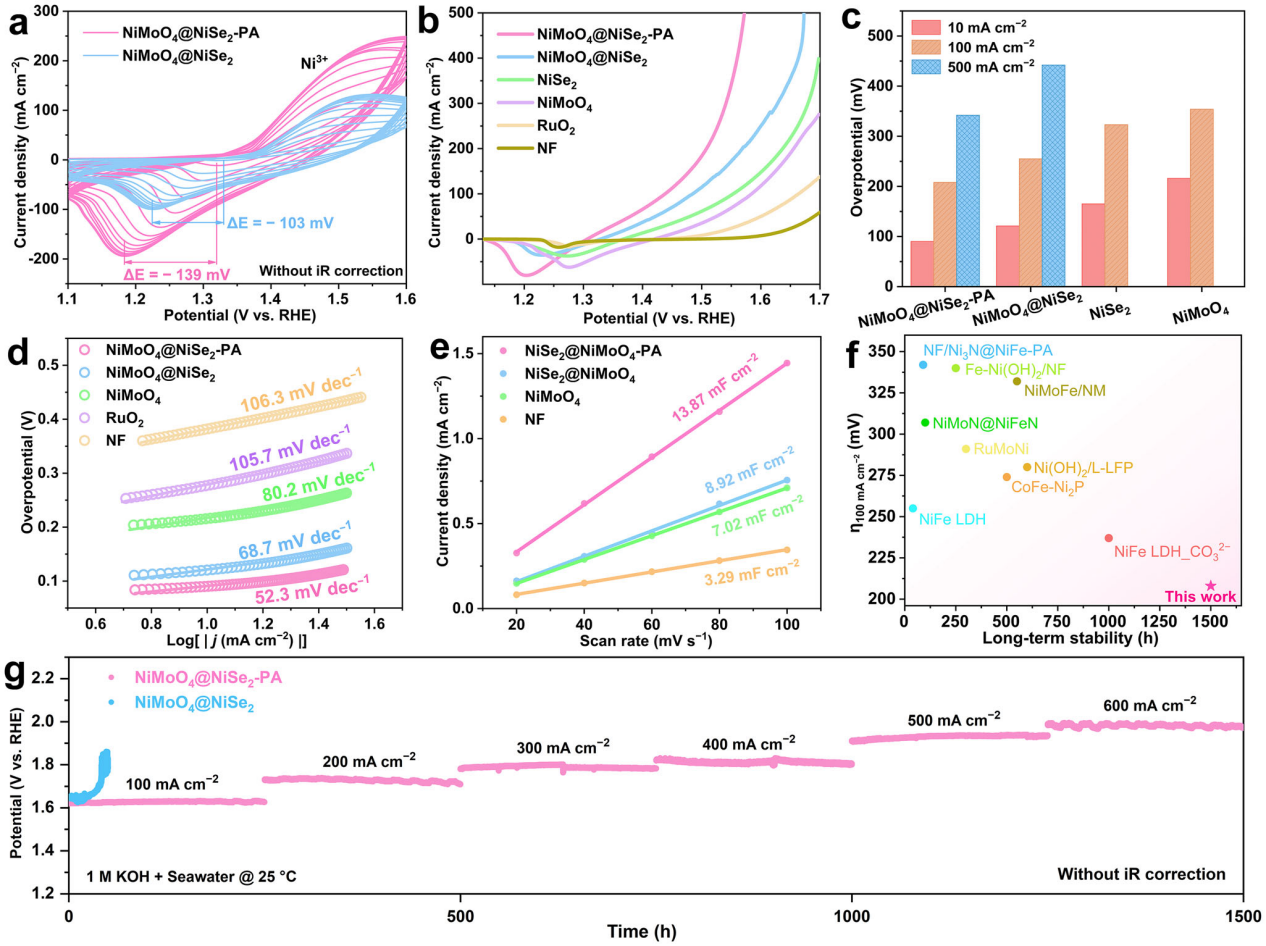
Electrolysis OER performance in 1.0 M KOH + seawater electrolyte at 25 °C. a) CV curves of NiMoO_4_@NiSe_2_‐PA and NiMoO_4_@NiSe_2_ electrocatalysts. b) Polarization curves of NiMoO_4_@NiSe_2_‐PA, NiMoO_4_@NiSe_2_, NiSe_2_, NiMoO_4_, RuO_2_, and NF at a scan rate of 5 mV s^−1^ with 85% iR correction. c) Comparison of overpotentials to achieve different current densities. d) C_dl_ plots, and e) Tafel curves of NiMoO_4_@NiSe_2_‐PA, NiMoO_4_@NiSe_2_, NiMoO_4_, and NF. f) Comparison of the overpotentials and durability of NiMoO_4_@NiSe_2_‐PA with other reported alkaline seawater OER catalysts. g) Long‐term durability of NiMoO_4_@NiSe_2_‐PA and NiMoO_4_@NiSe_2_ electrocatalyst at intermittent variable current density.

The electrochemical behavior of the NiMoO_4_@NiSe_2_‐PA electrocatalyst modified with surface PO_4_^3−^ groups was investigated using cyclic voltammetry (CV) in a solution of 1 m KOH + seawater (Seawater from Maidao Bay, as shown in Figure S7, Supporting Information). As shown in **Figure** **3**a, a redox peak pair was observed in the CV curves during the electrochemical reaction. It is worth noting that during CV cycling, the redox peak intensity and current continuously increase and gradually stabilize. It has been reported that the area of the redox peak pair is an important indicator of the catalytic activity of Ni‐based catalysts.^[^ ^48^ ^]^ Compared with NiMoO_4_@NiSe_2_, NiMoO_4_@NiSe_2_‐PA has a larger redox peak area, and the cathodic peak potential undergoes a much greater negative shift (ΔE = −139 mV) than NiMoO_4_@NiSe_2_ (ΔE = −103 mV). The results indicate that the modification of the surface PO_4_^3−^ groups facilitates the surface reconstruction of the catalyst, enabling the activation of more active sites at a lower potential (Ni^3+^).^[^ ^49^ ^]^ The catalytic activity of the NiMoO_4_@NiSe_2_‐PA electrocatalyst was evaluated using linear sweep voltammetry (LSV), as shown in Figure 3b and Figure S8 (Supporting Information). The NiMoO_4_@NiSe_2_‐PA electrocatalyst, modified with surface PO_4_^3−^ groups, can achieve a current density of 10 mA cm^−2^ with an overpotential of only 96 mV, which is significantly lower than those of NiMoO_4_@NiSe_2_ and commercial RuO_2_ catalysts. Moreover, the NiMoO_4_@NiSe_2_‐PA catalyst requires only 223 mV overpotential to achieve a high current density of 100 mA cm^−2^, which is significantly superior to other comparison catalysts (Figure 3c). This result demonstrates that the catalyst with PO_4_^3−^ group modification in the core‐shell nanowire structure exhibits excellent catalytic activity, thereby verifying the rationality of the structural design. Furthermore, the performance of this catalyst is superior to that of the currently reported alkaline seawater OER catalysts (Table S1, Supporting Information). In addition, NiMoO_4_@NiSe_2_‐PA has a lower Tafel slope (49.3 mV dec^−1^) and electrochemical impedance spectroscopy (1.1 Ω) compared to NiMoO_4_@NiSe_2_ (82.4 mV dec^−1^ and 1.7 Ω) and other comparison samples (Figure 3d; Figure S9, Supporting Information), indicating superior chemical kinetics and charge transfer ability of the catalyst. The electrochemically active surface area (ECSA) of the catalyst was evaluated based on the double‐layer capacitance (C_dl_), which was obtained from CV measurements at different scan rates (Figure S10, Supporting Information). As shown in Figure 3e, the C_dl_ value of NiMoO_4_@NiSe_2_‐PA is 13.87 mF cm^−2^, which is 35.7% higher than that of NiMoO_4_@NiSe_2_, indicating that the catalyst has more catalytically active sites. The intrinsic activity of the catalyst was obtained by normalizing the LSV curve by ECSA. NiMoO_4_@NiSe_2_‐PA consistently has optimal performance (Figure S11, Supporting Information), indicating excellent intrinsic catalytic activity of the catalyst. More importantly, the NiMoO_4_@NiSe_2_‐PA electrocatalyst has a long‐term stability of more than 1500 h at a continuous gradient current density (Figure 3f,g). Moreover, the catalytic activity remains undegraded at the industrial current density, demonstrating excellent application potential for industrial seawater electrolysis hydrogen production.

To further confirm the stability of the NiMoO_4_@NiSe_2_‐PA electrocatalyst, we conducted a series of characterizations on the samples after OER stability tests. SEM and STEM images reveal that the tested catalyst still has a core‐shell nanowire structure, indicating its robust structural stability (Figure S12, Supporting Information). TEM images showed that the nucleus retained its NiMoO_4_ crystal structure, and no continuous lattice striations were found in the shell, which may be due to the in situ reconstruction resulting in the formation of NiOOH species (Figure S13, Supporting Information). It is noteworthy that the surface PO_4_^3−^ layer remained stable after the stability test, which is crucial for repelling Cl^−^ during the reaction process. Furthermore, the EDS element mapping further demonstrates the stability of the catalyst's excellent structure and chemical composition. To further investigate the crystal structure after reconstruction, we performed XRD analysis following the stability test. As shown in Figure S14 (Supporting Information), the NiSe_2_ crystal phase of the NiMoO_4_@NiSe_2_‐PA catalyst disappeared after the stability test, confirming that the shell layer of the nanowires transformed into an amorphous structure during the structural reconstruction. In addition, the leaching amount of the element from the catalyst after the long‐term durability test was analyzed. Table S2 (Supporting Information) shows that the dissolution of active metal elements of NiMoO_4_@NiSe_2_‐PA (13.3 ug L^−1^) catalyst was significantly lower than that by NiMoO_4_@NiSe_2_ (2623.8 ug L^−1^) after testing. This strongly proves that the PO_4_^3−^ layer plays an important role in inhibiting metal dissolution. The changes in the surface chemical state of NiMoO_4_@NiSe_2_‐PA after durability tests were further investigated using high‐resolution XPS. As shown in Figure S15a (Supporting Information), the Ni *2p* XPS spectrum reveals that the Ni^3+^ peak shifts to a lower binding energy and exhibits increased intensity after the stability test, indicating a higher oxidation state of Ni in the catalyst.^[^ ^50^, ^51^ ^]^ This observation is consistent with the shift of the oxidation peak in the CV curve (Figure 3a), further confirming the structural reconstruction of the catalyst surface during the reaction, which plays a crucial role in enhancing catalytic activity and stability.^[^ ^52^, ^53^ ^]^ No obvious changes were observed in the XPS spectrum of Mo *3d*, indicating the excellent stability of the nuclear layer NiMoO_4_. However, the increase in the intensity of the SeO_x_ peak suggests that during the surface reconstruction process, Se may be oxidized to SeO_x_^2−^ (Figure S15b,c, Supporting Information). Notably, the PO_4_^3−^ groups remained stable after the OER durability test (Figure S15d, Supporting Information), which is consistent with the TEM analysis results, further confirming the surface PO_4_^3−^ layer's ability to prevent Cl^−^ inclusion.

### 2.3 Performance and Origin of Industrial‐Scale Seawater Hydrogen Production

**Figure 4:**
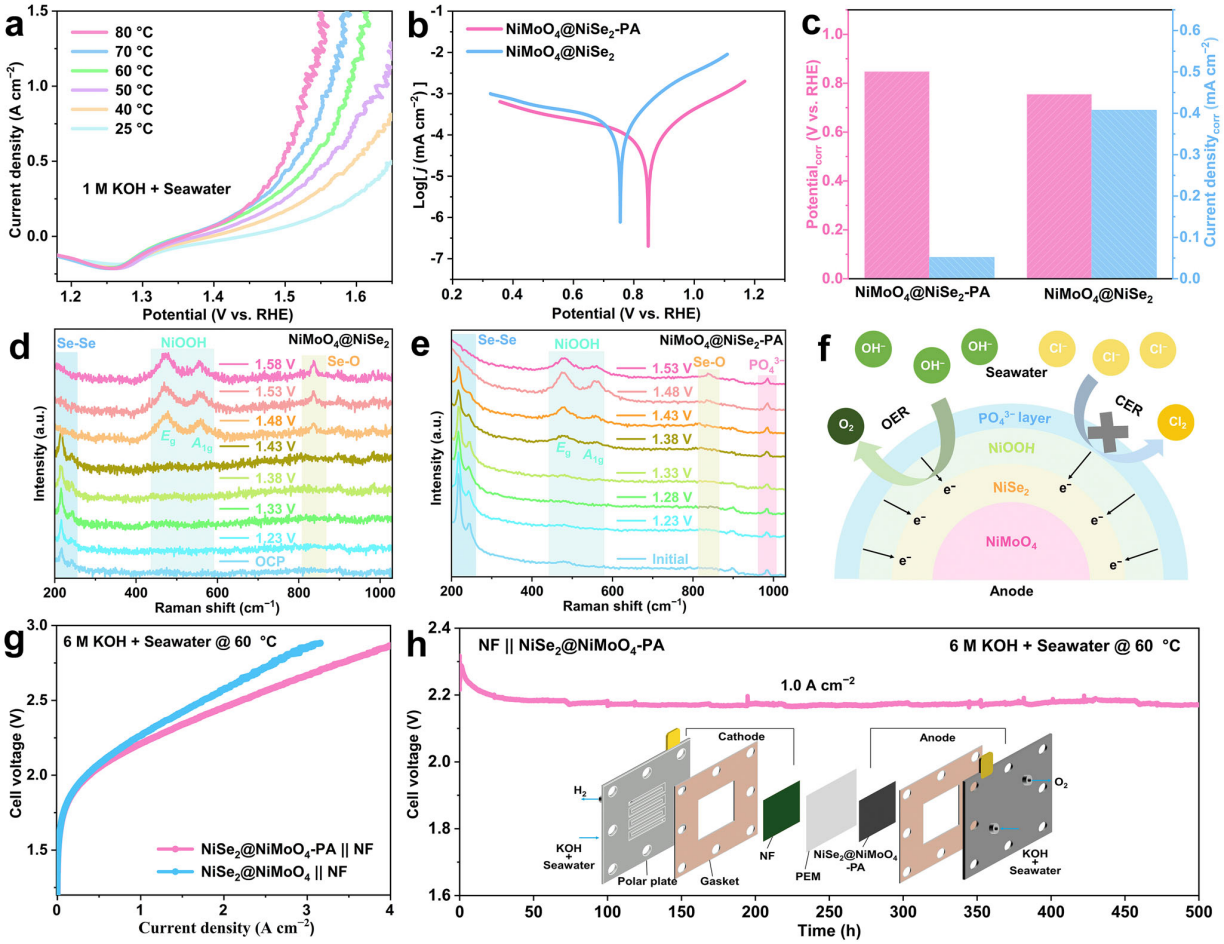
Performance and origin of industrial‐scale seawater hydrogen production. a) LSV curves of NiMoO_4_@NiSe_2_‐PA electrocatalysts at different temperatures. b) Electrochemical polarization curves of NiMoO_4_@NiSe_2_‐PA and NiMoO_4_@NiSe_2_ in alkaline seawater. c) Comparison of E_corr_ and I_corr_ of NiMoO_4_@NiSe_2_‐PA and NiMoO_4_@NiSe_2_. In situ Raman spectra in alkaline seawater for d) NiMoO_4_@NiSe_2_‐PA, and e) NiMoO_4_@NiSe_2_. f) Schematic diagram of Cl^−^ corrosion resistance and high‐performance mechanism of NiMoO_4_@NiSe_2_‐PA. g) Polarization curves without iR compensation of the NiMoO_4_@NiSe_2_‐PA || NF and NiMoO_4_@NiSe_2_ || NF couples operating. h) Long‐term durability test for NiMoO_4_@NiSe_2_‐PA || NF couples electrolyzer at 1.0 A cm^−2^ operated at 60 °C. The inset is a schematic diagram of the electrolyzer.

Considering the superior performance of NiMoO_4_@NiSe_2_‐PA electrocatalyst in alkaline seawater, we further evaluated its application potential for industrial‐level hydrogen production. As shown in **Figure** **4**a, the catalyst's performance gradually improves with increasing temperature, achieving a current density of 1.5 A cm^−2^ at a potential of only 1.55 V versus RHE at an industrial temperature (80 °C). Furthermore, in industrial‐level concentration alkaline seawater electrolyte (6 M KOH + seawater), the catalyst can achieve a current density of 2.5 A cm^−2^ at a potential of only 1.78 V versus RHE (Figure S16, Supporting Information). The corrosion resistance of the catalyst is crucial for industrial‐level seawater electrolysis for hydrogen production. Potentiodynamic polarization curves of NiMoO_4_@NiSe_2_‐PA and NiMoO_4_@NiSe_2_ electrocatalysts were tested at the open circuit potential in alkaline seawater electrolyte (Figure 4b; Figure S17, Supporting Information). As shown in Figure 4c, NiMoO_4_@NiSe_2_‐PA exhibits a higher corrosion potential (E_corr_ = 0.848 V) and lower corrosion current density (I_corr_ = 0.052 mA cm^−2^), indicating stronger corrosion resistance due to PO_4_^3−^ groups modification, consistent with long‐term durability test results.

To further explore the mechanism by which surface PO_4_^3−^ groups improve catalytic activity and anti‐chlorine corrosion, in situ Raman spectroscopic was used to characterize of the electrochemical reaction process of NiMoO_4_@NiSe_2_‐PA and NiMoO_4_@NiSe_2_ catalysts (Figure S18, Supporting Information). As shown in Figure 4d,e, in situ Raman spectroscopic indicates that the electrocatalyst undergoes surface reconstruction during the OER process. With the increase of the reaction potential, characteristic peaks appeared at ≈ 480 and 560 cm^−2^, which were respectively attributed to the bending and stretching vibrations of Ni−O in NiOOH.^[^ ^30^, ^54^ ^]^ This indicates that NiSe_2_ undergoes reconstitution during the OER reaction to form high‐valent Ni^3+^ in NiOOH as the actual active sites, which is consistent with the TEM and XPS results of the catalyst after the stability test. It is noteworthy that NiMoO_4_@NiSe_2_‐PA modified with PO_4_^3−^ groups began to undergo reconstruction at a potential of 1.38 V versus RHE, while NiMoO_4_@NiSe_2_ requires a potential of 1.48 V versus RHE. This indicates that the modification of the surface PO_4_^3−^ groups promotes the surface reconstruction of the electrocatalyst, thereby generating more active sites at a lower potential. More importantly, the characteristic peaks of PO_4_^3−^ groups remained almost unchanged during the OER reaction and reconstruction processes, indicating that the surface PO_4_^3−^ groups have super‐strong stability in improving catalytic activity and anti‐chlorine corrosion process (Figure 4e). Based on the above characterization results, the mechanism diagram of anti‐chlorine corrosion of NiMoO_4_@NiSe_2_‐PA electrocatalyst for high‐performance seawater electrolytic hydrogen production was obtained (Figure 4f). The porous NiMoO_4_@NiSe_2_ heterostructure facilitates electrolyte permeation and exposes more active sites. In addition, the modification of the surface PO_4_^3−^ groups not only promotes the PCET in the OER process, but also promotes the surface reconstruction of the shell NiSe_2_, thus accelerating the chemical kinetics and improving the catalytic activity. More importantly, the modification of the surface PO_4_^3−^ groups can effectively resist chlorine corrosion through electrostatic repulsion, resulting in excellent stability in the process of hydrogen production from seawater electrolysis.

To further demonstrate the potential of the NiMoO_4_@NiSe_2_‐PA electrocatalyst for industrial‐level seawater electrolysis to produce hydrogen, we assembled a proton exchange membrane electrolyzer, using NiMoO_4_@NiSe_2_‐PA as the anode and commercial NF as the cathode. As shown in Figure 4g, the optimal NiMoO_4_@NiSe_2_‐PA || NF exhibits superior performance in an industrial‐level setup, requiring only a voltage of 2.18 V to achieve a current density of 1 A cm^−2^. More importantly, the electrolyzer with the NiMoO_4_@NiSe_2_‐PA electrocatalyst as the anode showed strong long‐term durability at a current density of 1 A cm^−2^, and its performance showed no degradation over 500 h of operation (Figure 4h). The catalyst demonstrates superior performance and stability compared to recently reported electrolyzer catalysts (Table S3, Supporting Information), highlighting its potential for industrial‐scale seawater electrolysis for hydrogen production.

### 2.4 Density Functional Theory (DFT) Calculation

**Figure 5:**
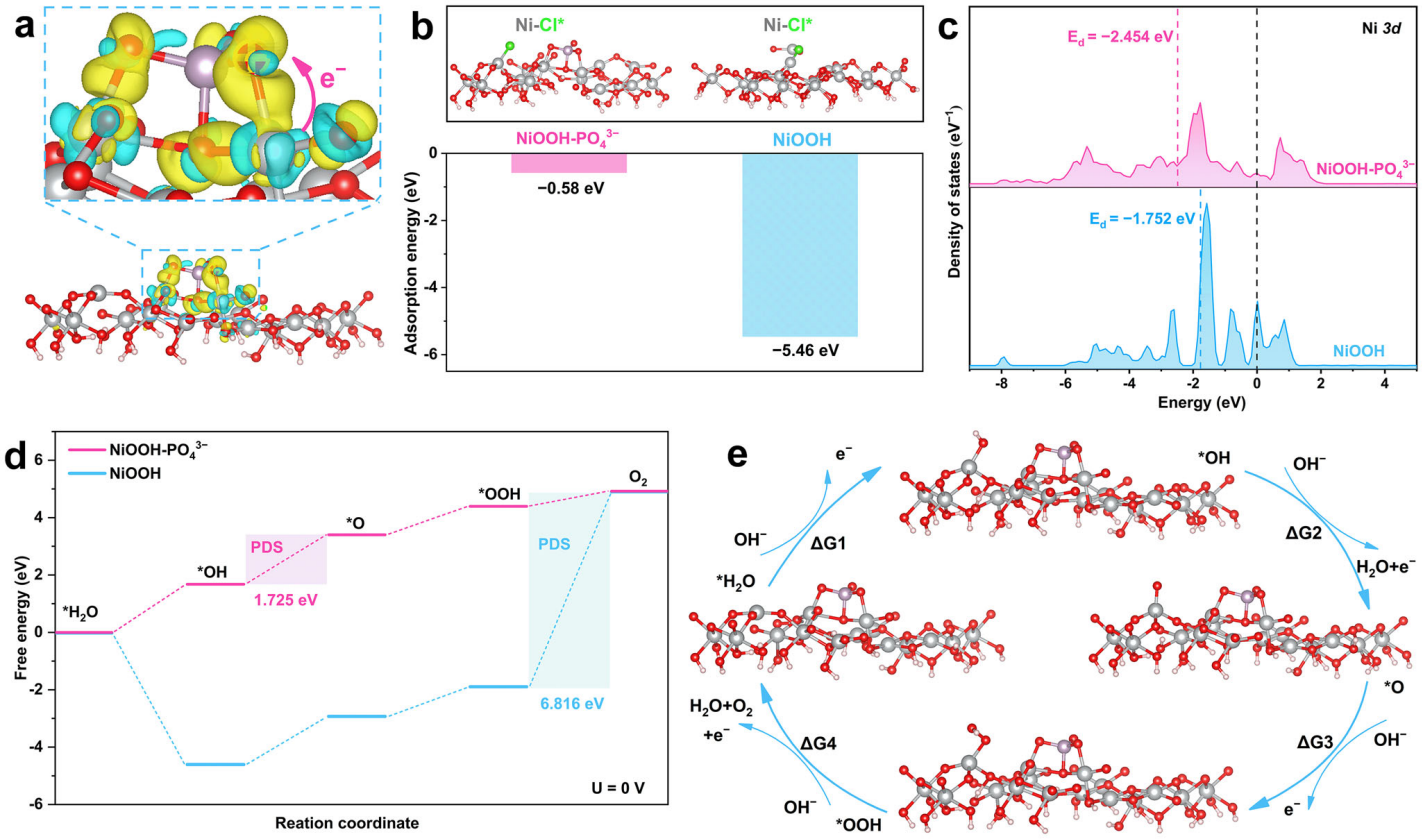
Anti‐chlorine corrosion and catalytic activity mechanism study using DFT calculation. a) Charge difference density of NiMoO_4_@NiSe_2_‐PA. b) Structural model and Ni─Cl^*^ adsorption energy for Cl^−^ ion adsorption on metal active sites. c) Density of states for NiMoO_4_@NiSe_2_‐PA and NiMoO_4_@NiSe_2_. d) Gibbs free energy diagram for NiMoO_4_@NiSe_2_‐PA and NiMoO_4_@NiSe_2_. e) Reaction pathway diagram for the four steps of the OER on NiMoO_4_@NiSe_2_‐PA.

Based on in situ Raman, HRTEM, and XPS analyses after durability tests, our analysis reveals that the surface reconstructed NiOOH is the real active site of the catalyst. To better understand the experimental results, we conducted DFT calculations to reveal the mechanism by which the modification of surface PO_4_^3−^ groups improves catalytic activity and anti‐chlorine corrosion. First, to more clearly elucidate the mechanism underlying the enhanced catalytic activity and corrosion resistance provided by surface PO_4_^3−^, we simplified the structural models of NiMoO_4_@NiSe_2_‐PA (NiOOH‐PO_4_^3−^) and NiMoO_4_@NiSe_2_ (NiOOH) after surface reconstruction (Figure S19, Supporting Information).^[^ ^53^ ^]^ The differential charge density map visually illustrates the redistribution of electrons between atoms, where yellow and cyan regions represent electron enrichment and depletion, respectively.^[^ ^55^ ^]^ **Figure** **5**a demonstrates significant charge transfer between NiOOH and PO_4_^3−^ groups, where electrons migrate from Ni atoms to PO_4_^3−^ groups, forming an electron‐rich region and thereby optimizing the electronic structure.^[^ ^56^, ^57^ ^]^ Furthermore, we evaluated the protective role of surface PO_4_^3−^ groups against chlorine corrosion by calculating the adsorption strength of Cl^−^ ions on the surface active metal of the catalyst. The adsorption energy of Cl^−^ ions on the Ni metal active sites on the surface of NiOOH is −5.46 eV, indicating that Cl^−^ ions has a strong affinity for the active metal sites (Figure 5b). In contrast, the adsorption energy of the catalyst modified by the surface PO_4_^3−^ groups for Cl^−^ ions is only −0.58 eV, which proves that the surface PO_4_^3−^ groups effectively inhibit the adsorption of Ni─Cl^*^, thus achieving the repulsion ability for Cl^−^ ions. This is the fundamental reason why this catalyst has outstanding corrosion resistance in seawater electrolysis for hydrogen production.

Density of states (DOS) analysis shows that the d‐band center of NiOOH is closer to the Fermi level, indicating that the adsorption of OH^*^ is excessively strong (Figure 5c).^[^ ^58^ ^]^ It is noteworthy that the d‐band center of NiOOH‐PO_4_^3−^, which is modified by the surface PO_4_^3−^ groups, is shifted away from the Fermi level. This implies that when d orbitals participate in bonding, more electrons are filled in the anti‐bonding orbitals, leading to lower adsorption energy and facilitating the desorption of intermediates.^[^ ^59^, ^60^ ^]^ The Gibbs free energy step diagrams illustrate that the rate‐determining steps of NiOOH is the ^*^OOH → O_2_ step with a corresponding theoretical potential of 5.586 V. This suggests that the OER pathway of NiOOH is constrained by the desorption of ^*^OOH intermediates, which aligns with the DOS analysis results. Interestingly, the PDS of the catalyst modified by the surface PO_4_^3−^ groups is transformed into the ^*^OH → ^*^O step, with a corresponding theoretical potential of only 0.495 V, indicating a lower energy barrier. (Figure 5d). Therefore, the modification of surface PO_4_^3−^ groups optimizes the electronic structure of the catalyst, making it more favorable for the adsorption and desorption of reaction intermediates (^*^OH, ^*^O, and ^*^OOH), thereby accelerating the chemical kinetics of water electrolysis (Figure 5e).

## 3 Conclusion

In conclusion, we proposed a surface amorphous PO_4_^3−^ layer modification to address the issues of active metal dissolution and chlorine corrosion of the catalyst in industrial‐scale seawater electrolysis for hydrogen production. A series of characterization results and DFT calculations demonstrates that the surface PO_4_^3−^ groups optimize the electronic structure of the catalyst by coordinating the electronic states. More importantly, the surface PO_4_^3−^ groups not only accelerate the in situ reconstruction of Ni to from NiOOH active sites, but also effectively inhibit the adsorption of chloride ions on the metal active sites, thereby enabling stable resistance to chlorine corrosion during seawater electrolysis for hydrogen production. In addition, as an anode catalyst for proton exchange membrane electrolyzers, a low cell voltage of 2.18 V is sufficient to reach the current density of 1 A cm^−2^, and the catalyst shows no degradation over 500 h of operation.

## Conflict of Interest

The authors declare no conflict of interest.

## Supporting information



Supporting Information

## Data Availability

The data that support the findings of this study are available in the supplementary material of this article.
