# Supplementary material for: Catalyst for Industrial‐Scale Seawater Electrolysis: Inhibit Active Metal Dissolution and Chlorine Corrosion
Source: Adv Sci (Weinh). 2025 Sep 16;12(45):e14301. doi: 10.1002/advs.202514301 (PMC12677663; doi:10.1002/advs.202514301)
Supplement: Supplementary file 1 — Supporting Information [file ADVS-12-e14301-s001.docx]

**Supplementary Information**

**Catalyst for Industrial-Scale Seawater Electrolysis: Inhibit Active Metal Dissolution and Chlorine Corrosion**

Peng Wang^1,†^, Jie Zheng^2,†^, Yuyang Li^1^, Qiaofu Shi^2^, Jun Zhang^1^, Yong Wan^1^, Mang Niu^3,*^, Yusuke Yamauchi^4,5,*^, Yun-Ze Long^1,*^

1 *Shandong Key Laboratory of Medical and Health Textile Materials, College of Physics, Qingdao University, Qingdao 266071, PR China*

2 *Industrial Research Institute of Nonwovens & Technical Textiles, Shandong Center for Engineered Nonwovens (SCEN), College of Textiles Clothing, Qingdao University, Qingdao 266071, PR China*

3 *State Key Laboratory of Bio-fibers and Eco-textiles, Institute of Biochemical Engineering, College of Materials Science and Engineering, Qingdao University, Qingdao 266071, PR China*

4 *School of Chemical Engineering, The University of Queensland, Brisbane, QLD 4072, Australia*

5 *Department of Materials Process Engineering, Graduate School of Engineering, Nagoya University, Nagoya 464-8603, Japan*

* Corresponding authors. E-mail addresses: mang.niu@qdu.edu.cn (M. Niu), y.yamauchi@uq.edu.au (Yusuke Yamauchi), yunze.long@qdu.edu.cn (Y. Z. Long).

† These two authors contributed equally to this work.

**Experimental Section**

**Preparation of precursor, NiMoO_4_, NiSe_2_, NiSe_2_@NiMoO_4_**

Under magnetic stirring, 0.352 g Na_2_MoO_4_·2H_2_O (≥99.95%, Aladdin), 0.582 g Ni(NO_3_)_2_·6H_2_O (≥99%, Aladdin), and 0.18 g urea (≥99.5%, Aladdin) were dissolved in 20 ml of deionized water. This mixed solution was then transferred into a 50 mL Teflon-lined stainless autoclave. Then a piece of cleaned Ni foam (3*3 cm^2^) was immersed into this solution, and the autoclave was maintained at 90 °C for 8 h to obtain NiMoO_4_ precursors. The NiSe_2_@NiMoO_4_ electrode was prepared by the chemical vapor deposition approach. Briefly, 0.5 g of selenium powder (≥99.9%, Aladdin) and the NiMoO_4_ precursors were placed on the upstream and the downstream sides of the tube furnace, respectively. The sample was then calcined at 350 °C for 3 h under an N_2_ atmosphere with a heating rate of 2 °C min^−1^. NiSe_2_ was synthesized by the same method without the participation of Na_2_MoO_4_·2H_2_O.

**Preparation of NiSe_2_@NiMoO_4_-PA electrode**

The surface PA-modified NiSe_2_@NiMoO_4_ nanoarray (NiSe_2_@NiMoO_4_-PA) was prepared by diluting 1 ml of a 50% (in H_2_O, Aladdin) phytic acid (PA) solution into 10 ml of ethanol and soaking the NiSe_2_@NiMoO_4_ electrode for 5 h.

**Physical characterization**

The morphological properties of the samples were observed using a field emission scanning electron microscope (FE-SEM, Regulus 8100, operated at 1 kV) and high-resolution transmission electron microscopy (HRTEM, JEM 2100F, operated at 200 kV). Energy-dispersive X-ray spectroscopy (EDX) mapping images were obtained using scanning transmission electron microscopy (STEM, Sigma 500) operated at an acceleration voltage of 300 kV. Data from inductively coupled plasma-optical emission spectrometry (ICP-OES) were obtained using the Thermo Fisher iCAP PRO instrument. X-ray diffraction (XRD) investigations were performed using a Smart Lab 3 KW diffractometer using Cu Kα (λ = 1.54056 Å). The chemical states of different elements were analyzed using X-ray photoelectron spectroscopy (XPS) using an ESCALAB Xi + X-ray photoelectron spectrometer. The binding energies were calibrated with C 1s peak (284.8 eV) as the reference. In-situ Raman spectroscopy was conducted on the IHR550, Horiba system under an excitation of 532 nm laser light (Renishaw), using a Hefei-made in-situ electrochemical cell (CIS-Raman-EC (S)–U1). The test potential for each measurement was controlled by an electrochemical workstation, with signals captured after 120 s of stabilization. The working, counter, and reference electrodes were the catalyst, a carbon rod, and an Hg/HgO electrode, respectively.

**Calculation method**

The DFT calculations were carried out using the Vienna Ab-initio Simulation Package (VASP) with the frozen-core all-electron projector-augment-wave (PAW) method.^[1,2]^ The Perdew-Burke-Ernzerhof (PBE) functional within the generalized gradient approximation (GGA) was adopted to describe the exchange and correlation potential.^[3]^ plane-wave basis set with a cutoff energy of 450 eV was used. A mono-layer 4×4 NiOOH (001) supercell was employed, and a 20 Å vacuum region was introduced above the supercell to minimize interlayer interactions between neighboring systems. A PO_4_ group was adsorbed onto the top layer of NiOOH (001) slab to construct the model of the NiOOH-PO_4_ composite. Geometry optimizations were carried out until the forces acting on each ion were reduced to less than 0.01 eV/Å, and a 1×1×1 Gamma Monkhorst-Pack k-point mesh was employed for sampling the Brillouin zone. The DFT-D3 method was used to describe the van der Waals interaction.^[4]^

The Gibbs free-energy (∆*G*) is calculated as follows:

∆*G* = *E*DFT + ∆*E*ZPE - *T*∆*S* (1)

where *E*_DFT_ is the total energy of the system calculated using DFT. ∆*E*_ZPE_ is the difference in zero-point energy between the adsorbed molecule and molecule in the gas phase, and ∆*S* is the entropy of one molecule between the absorbed state and the gas phase. *T* is the temperature (300 K). The adsorption energy (*E_ads_*) of Cl is calculated as follows:

*E_ads_*= *E_molecule+surface_* -*E_surface_* - *E_molecule_* (2)

where *E_surface_* is the energy of the NiOOH or NiOOH-PO_4 surface_, *E_molecule_* is the energy of a Cl atom, which is obtained by averaging the total energy of a Cl_2_ molecule. *E_molecule+surface_* represents the total energy of the adsorbed system.

**Electrochemical measurements**

Electrochemical measurements of all samples were performed using a standard three-electrode system at room temperature (25 °C) on an electrochemical workstation (CHI 660D, Chenhua, Shanghai). Hg/HgO electrode and carbon rod served as reference electrodes and counter electrodes, respectively, and 1 M KOH + Natural seawater were used as the electrolyte. Meanwhile, all the prepared samples were employed as the working electrodes. Linear sweep voltammetry (LSV) was employed to investigate the OER activity of the samples, with a scan rate of 5 mV s^-1^, and all the potentials were corrected with 90% iR compensation. All the potentials were converted to a reversible hydrogen electrode (RHE) using to the formula:

E_RHE_ = E_Hg/HgO_ + 0.098 + 0.0592 × pH (3)

The Tafel slopes were obtained by fitting the linear portion of the Tafel plots based on the equation:

η = b log(j) + a (4)

Cyclic voltammetry (CV) measurements were conducted at various scan rates (20, 40, 60, 80, and 100 mV s^-1^) to determine the double-layer capacitance (C_dl_) of the catalysts. The electrochemical active surface area (ECSA) was estimated using the equation:

ECSA = C_dl_/C_s_ (5)

Where C_s_ is the specific capacitance value of 0.04 mF cm^–2^. Electrochemical impedance spectra (EIS) measurements were performed over the frequency range from 0.01 kHz to 1000 kHz. To ensure that the OER reaction is in a stable catalytic state and to avoid interference from side reactions, we chose to measure EIS at a current density of 100 mA cm^−2^ (1.43 V vs. RHE). The long-term durability of the catalyst was assessed using chronopotentiometry (CP) at different current densities without iR compensation.

A serpentine flow-field dual-electrode flow cell (Wuhan Zhisheng New Energy Co.) was used as the reactor, where titanium plates served as bipolar plates. The synthesized catalyst was used as the anode, NF was selected as the cathode, and a proton exchange membrane (Nafion 117). Porous titanium mesh was used as the gas diffusion layer on both sides. The electrolyte, which consisted of 6 M KOH + seawater, was circulated at a flow rate of 50 mL min-1 using a peristaltic pump (BT100-2J). I-V curves were obtained through LSV at a scan rate of 10 mV s^-1^. The stability of the electrolyzer was evaluated under chronopotentiometry (CP) testing conditions.


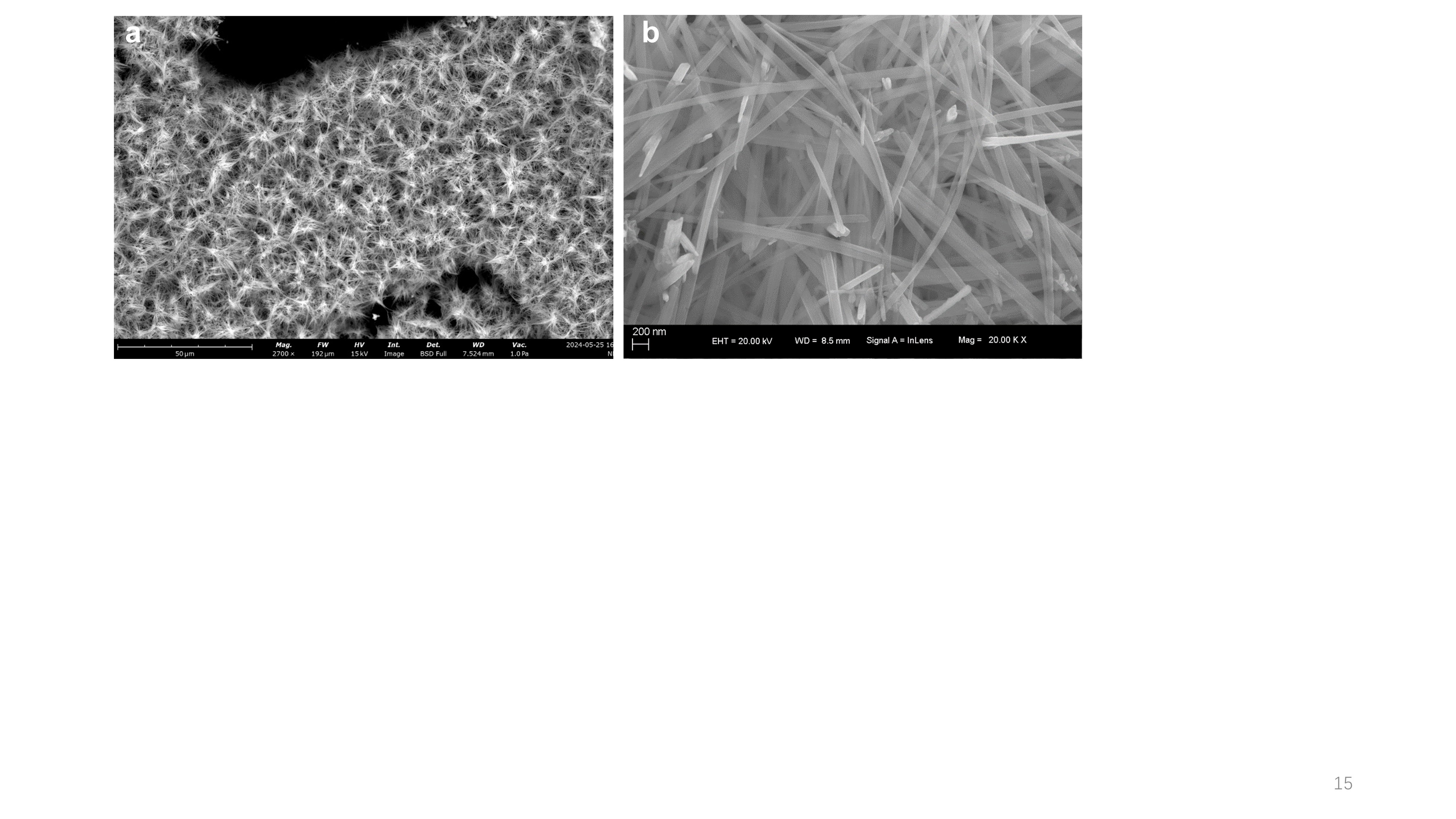


**Figure S1.** a,b) SEM images of the NiMoO_4_ precursor.


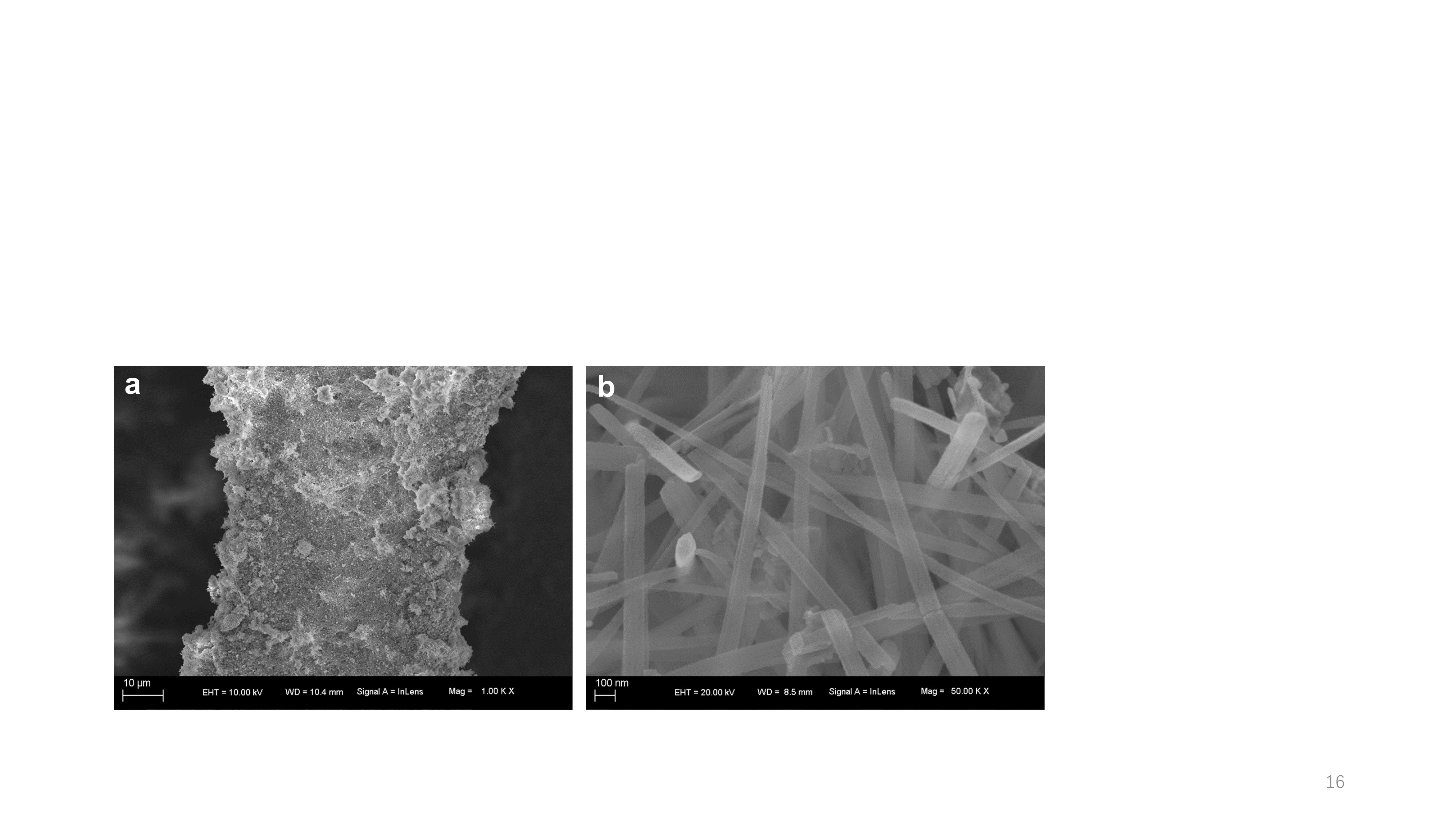


**Figure S2.** a,b) SEM images of the NiMoO_4_@NiSe_2_ electrocatalyst.


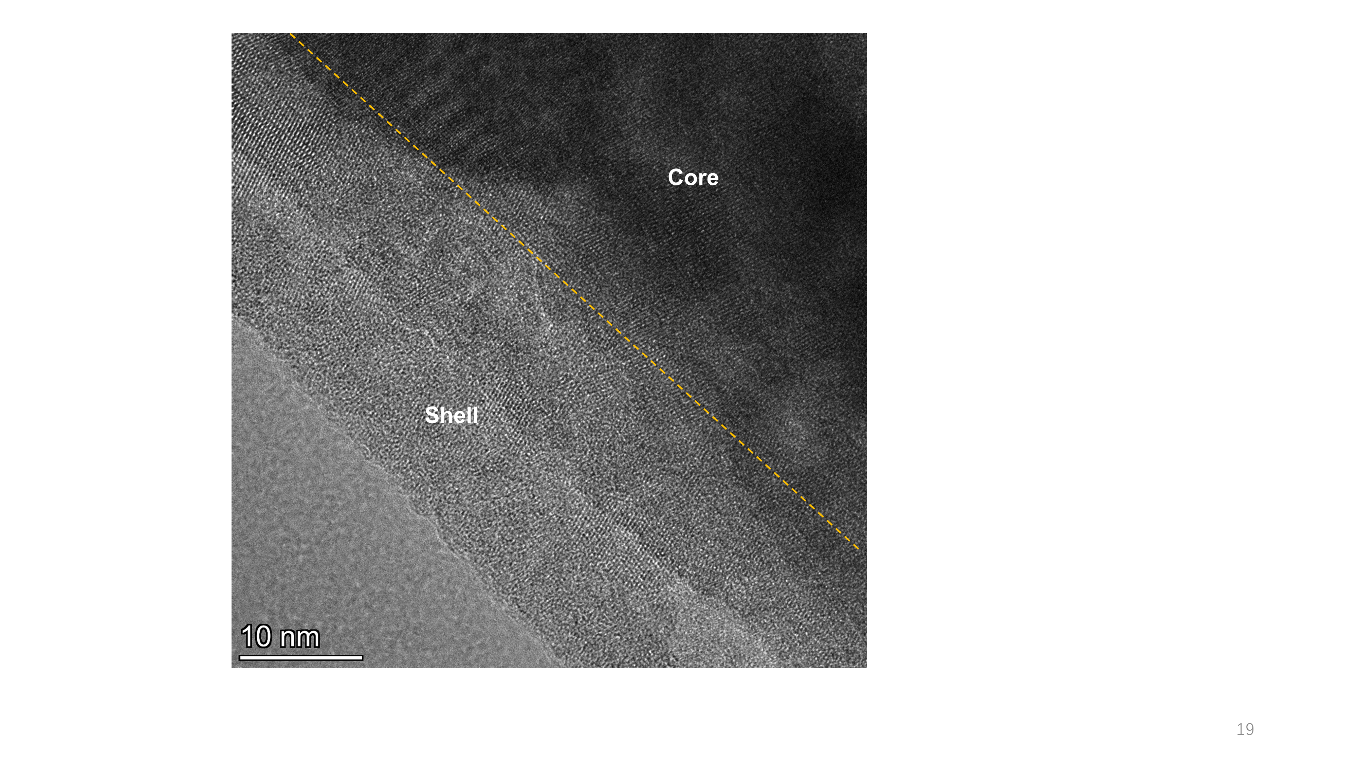


**Figure S3.** HRTEM image of the NiMoO_4_@NiSe_2_-PA core-shell nanowire.


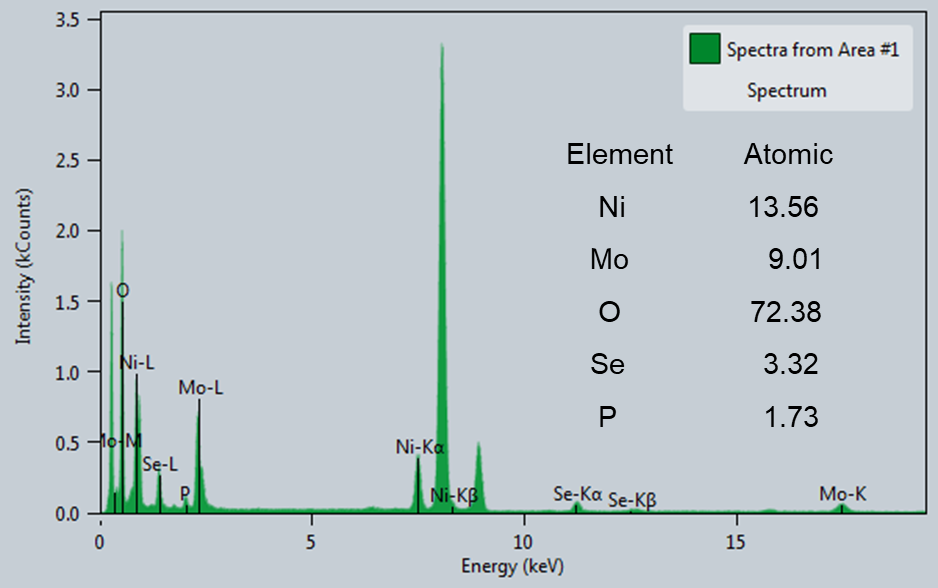


**Figure S4.** HAADF-STEM-EDS spectrum of NiMoO_4_@NiSe_2_-PA.


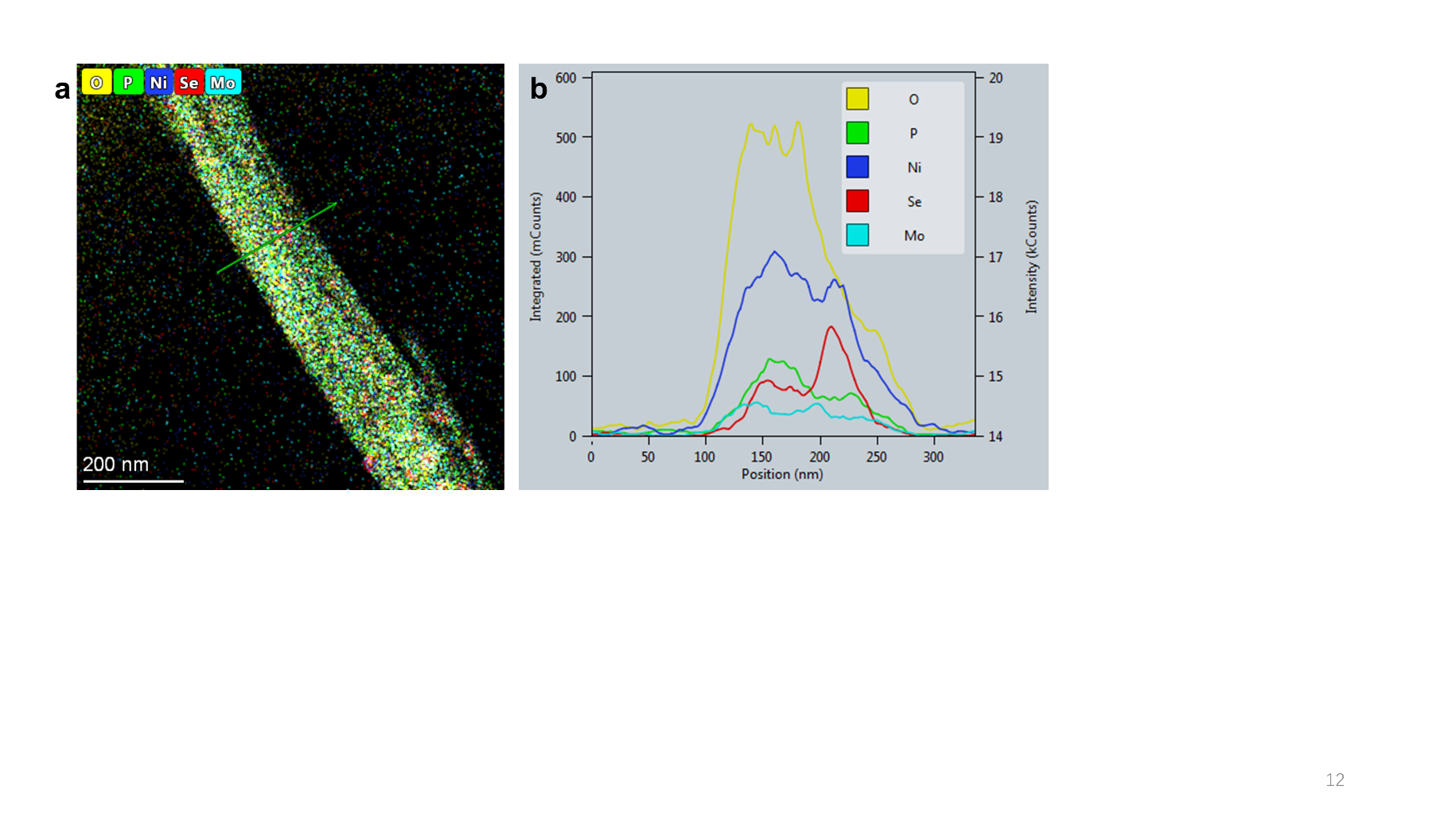


**Figure S5.** (a) HAADF-STEM image of NiMoO_4_@NiSe_2_-PA and corresponding EDS element mappings of Ni, Mo, O, Se, and P. (b) Linear element distribution of NiMoO_4_@NiSe_2_-PA core-shell nanorods.





**Figure S6.** XPS survey spectrum of NiMoO_4_@NiSe_2_-PA.


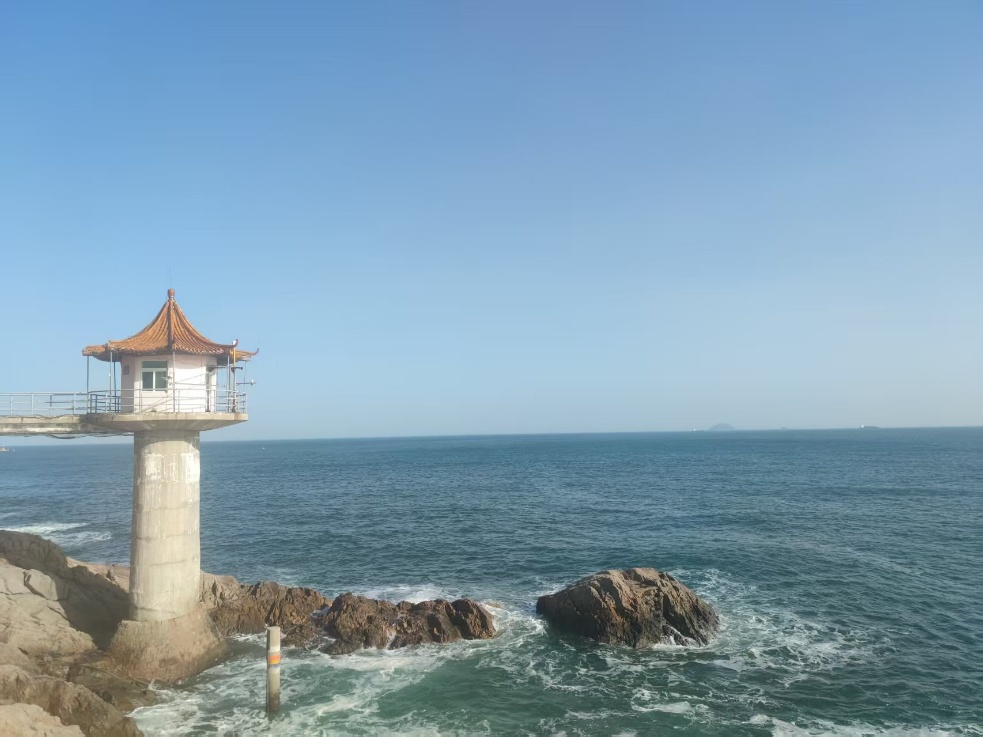


**Figure S7** Photo of Maidao Bay, Qingdao. All seawater used in our manuscript was from southeast coast of the Yellow Sea of China, located at 36°3'48'' north latitude and 120°25'14'' east longitude. The natural seawater was pretreated before use, and the large suspended solids and microorganisms in seawater were removed by physical filtration to reduce the interference to the electrolysis process.





**Figure S8.** Polarization curves of NiMoO_4_@NiSe_2_-PA, NiMoO_4_@NiSe_2_, NiMoO_4_, RuO_2_, and NF at a scan rate of 5 mV s^−1^ witout iR correction.





**Figure S9.** Nyquist plots of the NiMoO_4_@NiSe_2_-PA, NiMoO_4_@NiSe_2_, NiMoO_4_, RuO_2_, and NF.





**Figure S10.** CV curves in the non-Faraday region with different sweep speeds of the a) NiMoO_4_@NiSe_2_-PA, b) NiMoO_4_@NiSe_2_, c) NiMoO_4_, and d) NF.





**Figure S11.** Normalized OER performance of electrocatalyst. a) The electrochemically active surface area (ECSA) and b) LSV curves normalized to ECSA for NiMoO_4_@NiSe_2_-PA, NiMoO_4_@NiSe_2_, NiMoO_4_, and NF in 1.0 M KOH +seawater solution.


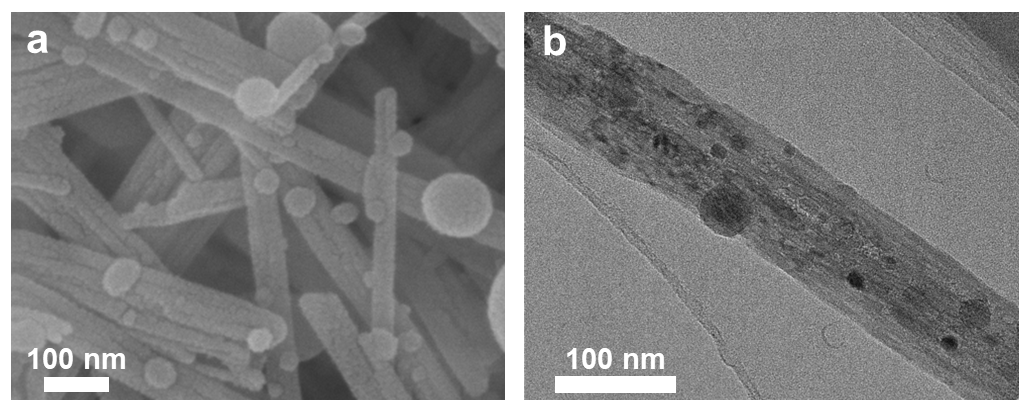


**Figure S12.** a) SEM and b) STEM image of the NiMoO_4_@NiSe_2_-PA after OER stability testing in alkaline seawater.


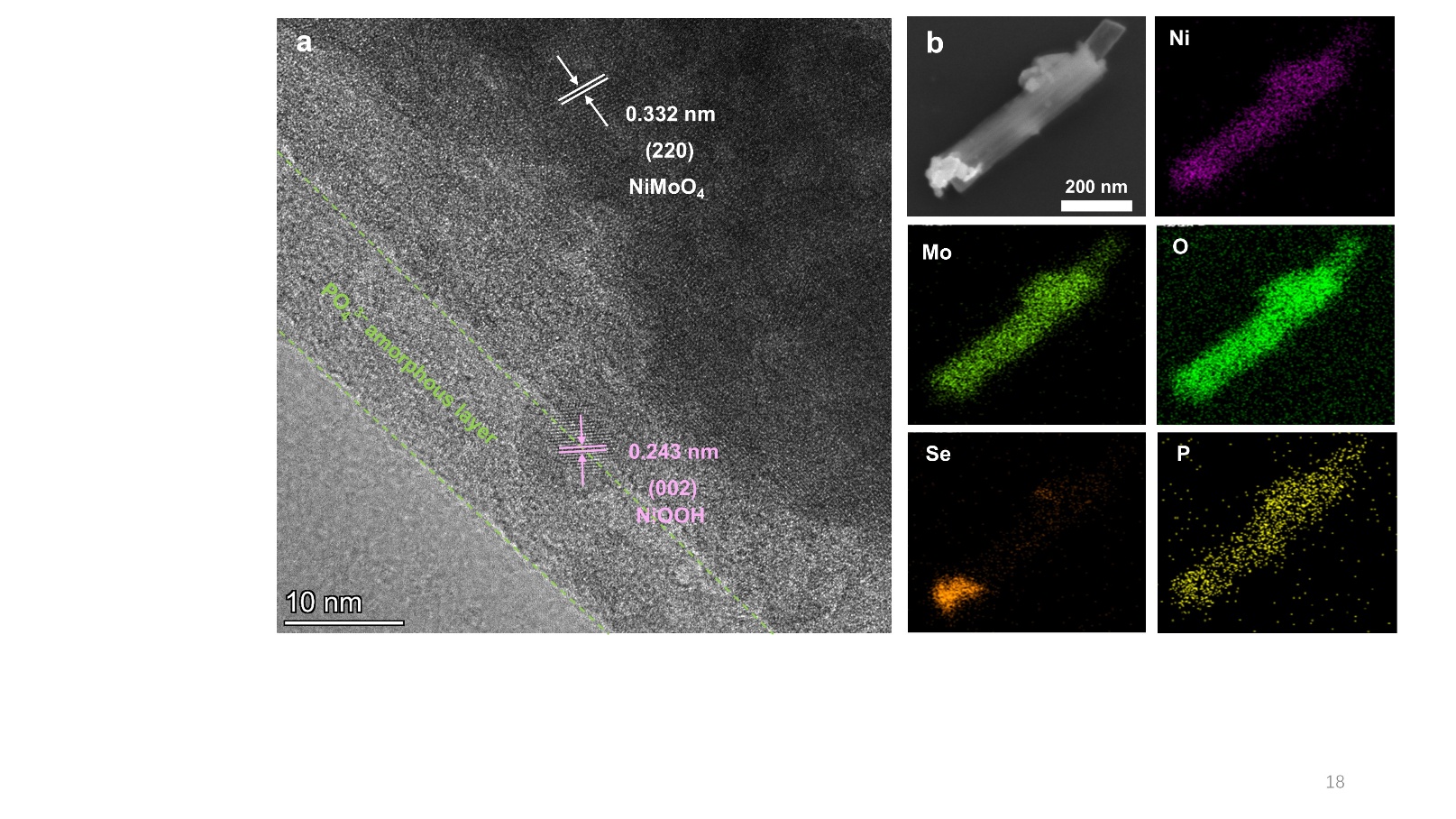


**Figure S13.** (**a**) TEM and (**b**) corresponding EDS elemental mapping of the NiMoO_4_@NiSe_2_-PA after OER stability testing in alkaline seawater.





**Figure S14.** XRD patterns of the NiMoO_4_@NiSe_2_-PA after OER stability testing in alkaline seawater.





**Figure S15.** High-resolution X-ray photoelectron spectroscopy (XPS) spectra of elements a) Ni, b) Mo, c) Se, and d) P of NiMoO_4_@NiSe_2_-PA after durability testing in alkaline seawater.





**Figure S16.** LSV curves of NiMoO_4_@NiSe_2_-PA electrocatalysts with different electrolytes.





**Figure S17.** OCP measurement of NiMoO_4_@NiSe_2_-PA and NiMoO_4_@NiSe_2_ in alkaline seawater.


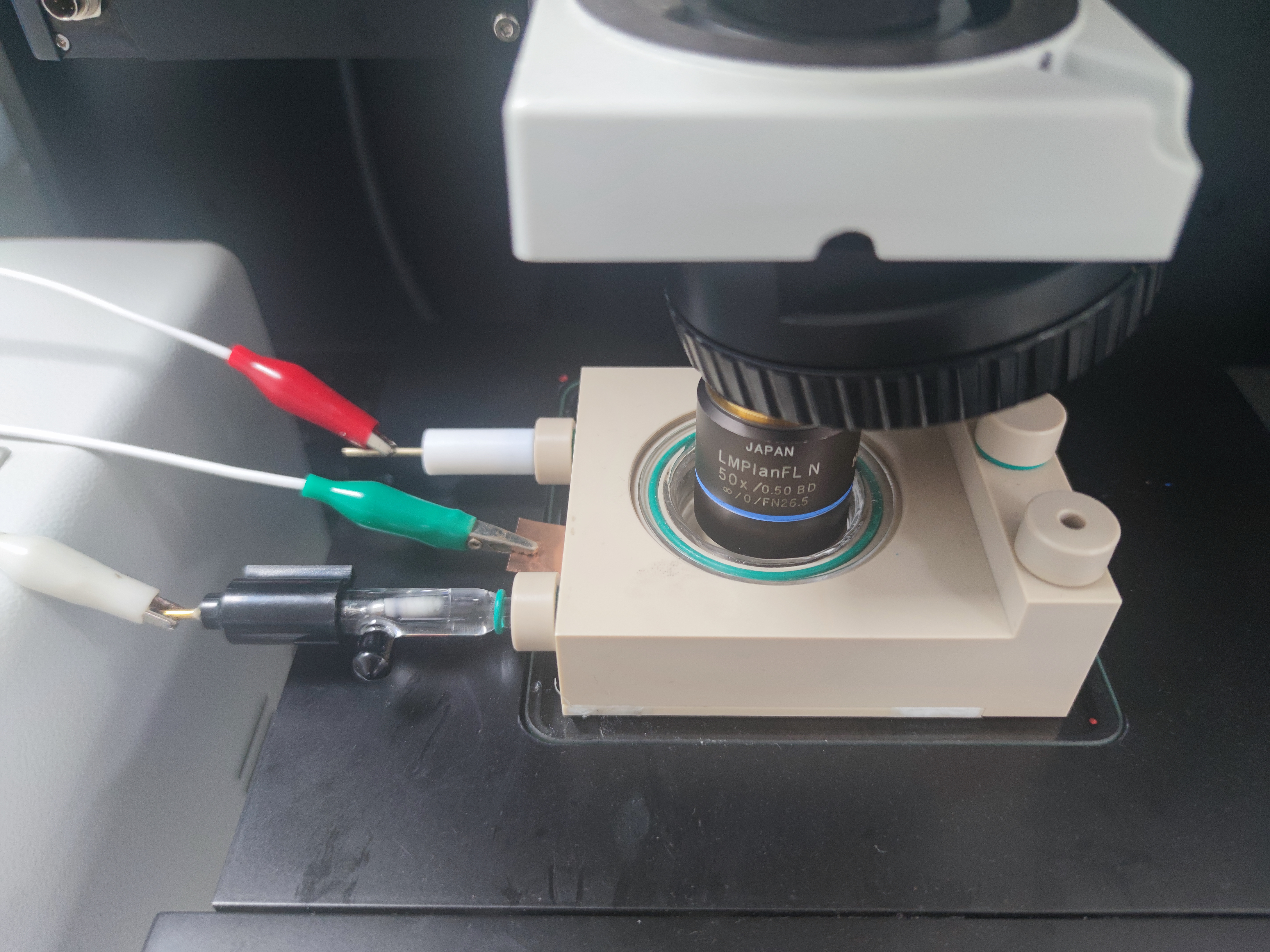


Optical photograph of the in-situ Raman test setup.

**Figure S18.** OCP measurement of NiMoO_4_@NiSe_2_-PA and NiMoO_4_@NiSe_2_ in alkaline seawater.





**Figure S19.** Optimization of the NiMoO_4_@NiSe_2_-PA structure model

**Table S1.** Comparison of OER performance for the currently reported OER catalysts.

| **Electrocatalysts** | **Electrolyte** | **η_100_ for OER (mV)** | **Stability (h)** | **Ref.** |
| --- | --- | --- | --- | --- |
| NiMoO_4_@NiSe_2_-PA | 1 M KOH +  seawater | 208 | 1500 | **This work** |
| Fe-Ni(OH)_2_/NF | 4 M KOH +  seawater | 340 | 250 | ^[5]^ |
| FeMoOOH/NF | 1 M KOH +  seawater | 240 | 1000 | ^[6]^ |
| caMo-NiFePO/NMF | 1 M KOH +  seawater | 259 | 120 | ^[7]^ |
| NiFe LDH_CO_3_^2−^ | 1 M KOH + 0.5 M NaCl | 237 | 1000 | ^[8]^ |
| LiFePO_4_ (Ni(OH)_2_/L-LFP) | 1 M KOH +  seawater | 280 | 600 | ^[9]^ |
| RuMoNi | 1 M KOH +  seawater | 291 | 300 | ^[10]^ |
| NiMoFe/NM | 1 M KOH +  seawater | 332 | 550 | ^[11]^ |
| NF/Ni_3_N@NiFe-PA | 1 M KOH + 0.5 M NaCl | 342 | 90 | ^[12]^ |
| Ni(OH)_2_-TCNQ/GP | 1 M KOH + 0.5 M seawater | 382 | 80 | ^[13]^ |
| NiMoN@NiFeN | 1 M KOH +  seawater | 307 | 100 | ^[14]^ |
| CoFe-Ni_2_P | 1 M KOH +  seawater | 274 | 500 | ^[15]^ |
| CoFe-Ci@GQDs/NF | 1 M KOH + 0.5 M NaCl | 255 | 2800 | ^[16]^ |
| Fe_2_P/Ni_1.5_Co_1.5_N/N_2_P | 1 M KOH +  seawater | 255 | 40 | ^[17]^ |
| S-(Ni,Fe)OOH | 1 M KOH +  seawater | 281 | 100 | ^[18]^ |
| Cr-Co_x_P | 1 M KOH +  seawater | 325 | 140 | ^[19]^ |
| F-FeCoPv@IF | 1 M KOH +  0.5 NaCl | - | 20 | ^[20]^ |
| Ni_x_Cr_y_O | 1 M KOH +  seawater | 370 | 275 | ^[21]^ |
| NiFeCo-LDH | 1 M KOH +  seawater | 304 | 80 | ^[22]^ |
| B, Fe-CoP | 1 M KOH +  seawater | 282 | 200 | ^[23]^ |
| Fe-Ni_2_P_v_ | 1 M KOH +  seawater | 180 | 100 | ^[24]^ |
| NiFe LDH | 1 M KOH + 0.5 M NaCl | 254 | 300 | ^[25]^ |

**Table S2.** Elemental contents of Mo, Ni, Se and P in electrolyte after long-term durability tests was tested using inductively coupled plasma mass spectrometry (ICP-MS).

| **Electrocatalysts** | **Element** | **Contents (ug L^-1^)** |
| --- | --- | --- |
| NiMoO_4_@NiSe_2_-PA | Ni | 13.3 |
|  | Mo | 2028.3 |
|  | Se | 1741.5 |
|  | P | 138.5 |
| NiMoO_4_@NiSe_2_ | Ni | 2623.8 |
|  | Mo | 3225.2 |
|  | Se | 3445.6 |

**Table S3.** Comparison of the performance and stability of recently reported catalysts for two-electrode system electrolyzers.

| **Electrocatalysts** | **Membrane** | **Electrolyte** | **T (°C)** | **Performance** | | **Stability** | **Ref.** |
| --- | --- | --- | --- | --- | --- | --- | --- |
| NiMoO_4_@NiSe_2_-PA\|\|NF | PEM, Nafion 117 | 6 M KOH + seawater | 60 | | 2.18 V@ 1 A cm^−2^ | 500 | **This work** |
| NiFe LDH-[PO_4_^3−^] \|\| NF | AWE, polymer | 6 M KOH + seawater | 80 | | 2.0 V@ 0.5 A cm^−2^ | 100 | ^[26]^ |
| Cr_2_O_3_–CoO_x_\|\|TF | PEM, Nafion 115 | seawater | 25 | | 2.33 V@ 1 A cm^−2^ | 100 | ^[27]^ |
| Ni_3_FeN@PO_4_^3−^\|\|Pt | AEM, FAB-PK-130 | 6 M KOH + seawater | 25 | | 1.95 V@ 0.5 A cm^−2^ | 200 | ^[28]^ |
| Y-NiMo/MoO_2−x_\|\|NiFe LDH | AEM, X37-50 Grade T | 3 M KOH + seawater | 25 | | 1.82 V@ 0.5 A cm^−2^ | 220 | ^[29]^ |
| CoFeAl-LDH\|\|NF | AEM, Zirfon UPT | 20wt.% NaOH + 6-fold  concentrated seawater | 80 | | 2.06 V@ 1 A cm^−2^ | 500 | ^[30]^ |
| CoFe-Ni_2_P/NF | AEM, FAA-3-PK-130 | 6 M KOH + seawater | -- | | 2.25 V@ 1 A cm^−2^ | 350 | ^[15]^ |
| RuMoNi\|\|RuMoNi | AEM, X37-50 Grade T | 1 M KOH + seawater | 60 | | 1.85 V@ 0.5 A cm^−2^ | 240 | ^[10]^ |
| Ni-MoN\|\|CF | AEM, FAA-3-PK-130 | 1 M KOH + seawater | 60 | | 1.8 V@ 0.5 A cm^−2^ | 100 | ^[31]^ |
| SOM-Ni-Fe \|\|NiMo | PPS | 1 M KOH + seawater | 65 | | ~2.8 V@ 1.3 A cm^−2^ | 220 | ^[32]^ |
| NiFeLDH-FLPs | AEM | 1 M KOH + seawater | 60 | | ~1.55 V@ 0.2 A cm^−2^ | 250 | ^[33]^ |
| NiFeP/NiS-A\|\|Pt/C | AEM, Sustainion®X37-50 | 0.5 M KHCO_3_+seawater | 25 | | 2.08 V@ 1 A cm^−2^ | 220 | ^[34]^ |

**References**

[1] G. Kresse, J. Hafner, *Phys. Rev. B* **1994**, *49*, 14251-14269.

[2] G. Kresse, J. Furthmuller, *Phys. Rev. B* **1996**, *54*, 11169-11186.

[3] G. C. Yang, Y. Q. Jiao, H. J. Yan, Y. Xie, C. G. Tian, A. P. Wu, Y. Wang, H. G. Fu, *Nat. Commun.* **2022**, *13*, 3125.

[4] S. Grimme, J. Antony, S. Ehrlich, H. Krieg, *J. Chem. Phys.* **2010**, *132*, 154104.

[5] T. Liu, C. Lan, M. Tang, M. X. Li, Y. T. Xu, H. R. Yang, Q. Y. Deng, W. C. Jiang, Z. Y. Zhao, Y. F. Wu, H. P. Xie, *Nature Communications* **2024**, *15*, 8874.

[6] J. P. Sun, S. Zhou, Z. Zhao, S. Y. Qin, X. C. Meng, C. H. Tung, L. Z. Wu, *Energy & Environmental Science* **2025**, *18*, 1952-1962.

[7] P. F. Tian, W. Zong, J. Xiong, W. Liu, J. Q. Liu, Y. H. Dai, J. X. Zhu, S. T. Huang, S. W. Song, K. B. Chu, G. J. He, N. Han, *Advanced Functional Materials* **2025**, 2504862.

[8] P. J. Deng, Y. Liu, H. L. Liu, X. A. Li, J. J. Lu, S. Y. Jing, P. Tsiakaras, *Advanced Energy Materials* **2024**, *14*, 2400053.

[9] Z. Li, M. T. Li, Y. Q. Chen, X. C. Ye, M. J. Liu, L. Y. S. Lee, *Angewandte Chemie-International Edition* **2024**, *63*, e202410396.

[10] X. Kang, F. N. Yang, Z. Y. Zhang, H. M. Liu, S. Y. Ge, S. Q. Hu, S. H. Li, Y. T. Luo, Q. M. Yu, Z. B. Liu, Q. Wang, W. C. Ren, C. H. Sun, H. M. Cheng, B. L. Liu, *Nature Communications* **2023**, *14*, 3607.

[11] L. Shao, X. D. Han, L. Shi, T. Z. Wang, Y. S. Zhang, Z. Q. Jiang, Z. X. Yin, X. R. Zheng, J. H. Li, X. P. Han, Y. D. Deng, *Advanced Energy Materials* **2024**, *14*.

[12] P. Li, S. Zhao, Y. Q. Huang, Q. H. Huang, B. J. Xi, X. G. An, S. L. Xiong, *Advanced Energy Materials* **2024**, *14*, 2303360.

[13] L. C. Zhang, J. Q. Wang, P. Y. Liu, J. Liang, Y. S. Luo, G. W. Cui, B. Tang, Q. Liu, X. D. Yan, H. G. Hao, M. L. Liu, R. Gao, X. P. Sun, *Nano Research* **2022**, *15*, 6084-6090.

[14] L. Yu, Q. Zhu, S. W. Song, B. McElhenny, D. Z. Wang, C. Z. Wu, Z. J. Qin, J. M. Bao, Y. Yu, S. Chen, Z. F. Ren, *Nature Communications* **2019**, *10*, 5106.

[15] C. Q. Huang, Q. C. Zhou, L. Yu, D. S. Duan, T. Y. Cao, S. H. Qiu, Z. Z. Wang, J. Guo, Y. X. Xie, L. P. Li, Y. Yu, *Advanced Energy Materials* **2023**, *13*, 2301475.

[16] R. L. Fan, C. H. Liu, Z. H. Li, H. T. Huang, J. Y. Feng, Z. S. Li, Z. G. Zou, *Nature Sustainability* **2024**, *7*, 158−167.

[17] F. M. Zhang, Y. L. Liu, F. Yu, H. J. Pang, X. Zhou, D. Y. Li, W. Q. Ma, Q. Zhou, Y. X. Mo, H. Q. Zhou, *Acs Nano* **2023**, *17*, 1681-1692.

[18] L. Yu, L. B. Wu, B. McElhenny, S. W. Song, D. Luo, F. H. Zhang, Y. Yu, S. Chen, Z. F. Ren, *Energy & Environmental Science* **2020**, *13*, 3439-3446.

[19] Y. Y. Song, M. Z. Sun, S. C. Zhang, X. Y. Zhang, P. Yi, J. Z. Liu, B. L. Huang, M. H. Huang, L. X. Zhang, *Advanced Functional Materials* **2023**, *33*.

[20] J. W. Zhu, J. Q. Chi, T. Cui, L. L. Guo, S. Q. Wu, B. Li, J. P. Lai, L. Wang, *Applied Catalysis B-Environment and Energy* **2023**, *328*, 122487.

[21] A. Malek, Y. R. Xue, X. Lu, *Angewandte Chemie-International Edition* **2023**, *62*, e202309854.

[22] Y. S. Park, J. Y. Jeong, M. J. Jang, C. Y. Kwon, G. H. Kim, J. Jeong, J. H. Lee, J. Y. Lee, S. M. Choi, *Journal of Energy Chemistry* **2022**, *75*, 127-134.

[23] Y. J. Pan, Z. C. Wang, K. X. Wang, Q. Ye, B. S. Shen, F. S. Yang, Y. L. Cheng, *Advanced Functional Materials* **2024**, *34*, 2402264.

[24] X. B. Liu, Q. P. Yu, X. Y. Qu, X. P. Wang, J. Q. Chi, L. Wang, *Advanced Materials* **2024**, *36*, 2307395.

[25] H. Liu, W. Shen, H. Y. Jin, J. Xu, P. X. Xi, J. C. Dong, Y. Zheng, S. Z. Qiao, *Angewandte Chemie-International Edition* **2023**, *62*, e202311674.

[26] X. G. Sun, W. Shen, H. Liu, P. X. Xi, M. Jaroniec, Y. Zheng, S. Z. Qiao, *Nature Communications* **2024**, *15*, 10351.

[27] J. X. Guo, Y. Zheng, Z. P. Hu, C. Y. Zheng, J. Mao, K. Du, M. Jaroniec, S. Z. Qiao, T. Ling, *Nature Energy* **2023**, *8*, 264-272.

[28] H. S. Hu, X. L. Wang, Z. R. Zhang, J. H. Liu, X. H. Yan, X. L. Wang, J. C. Wang, J. P. Attfield, M. H. Yang, *Adv. Mater.* **2025**, *37*, 2415421.

[29] S. J. Liu, Z. G. Zhang, K. Dastafkan, Y. Shen, C. Zhao, M. K. Wang, *Nature Communications* **2025**, *16*, 773.

[30] W. Liu, J. G. Yu, T. S. Li, S. H. Li, B. Y. Ding, X. L. Guo, A. Q. Cao, Q. H. Sha, D. J. Zhou, Y. Kuang, X. M. Sun, *Nature Communications* **2024**, *15*, 4712.

[31] L. B. Wu, F. H. Zhang, S. W. Song, M. H. Ning, Q. Zhu, J. Q. Zhou, G. H. Gao, Z. Y. Chen, Q. C. Zhou, X. X. Xing, T. Tong, Y. Yao, J. M. Bao, L. Yu, S. Chen, Z. F. Ren, *Advanced Materials* **2022**, *34*, 2201774.

[32] Q. Niu, F. Y. Gao, X. G. Sun, Y. Zheng, S. Z. Qiao, *Advanced Functional Materials* **2025**, 2504872.

[33] J. W. Zhu, T. Cui, J. Q. Chi, T. T. Wang, L. L. Guo, X. B. Liu, Z. X. Wu, J. P. Lai, L. Wang, *Angewandte Chemie-International Edition* **2025**, *64*, e202414721.

[34] M. Han, H. B. Wang, J. S. Zhou, K. N. Liu, N. Wang, X. H. Chen, Y. C. Liu, H. Y. Liang, *Advanced Functional Materials* **2025**, *35*, 2415143.
